# Facilitation of motor adaptation using multiple gait rehabilitation interventions

**DOI:** 10.3389/fresc.2024.1238139

**Published:** 2024-10-10

**Authors:** Adila Hoque, Seok Hun Kim, Kyle B. Reed

**Affiliations:** ^1^ REEDlab, Department of Mechanical Engineering, University of South Florida, Tampa, FL, United States; ^2^ School of Physical Therapy and Rehabilitation Sciences, University of South Florida, Tampa, FL, United States

**Keywords:** split-belt treadmill, rhythmic auditory cueing, learning rate, spatiotemporal asymmetry, sensorimotor adaptation, generalization, working memory

## Abstract

**Introduction:**

The rate of adjustment in a movement, driven by feedback error, is referred to as the adaptation rate, and the rate of recovery of a newly adapted movement to its unperturbed condition is called the de-adaptation rate. The rates of adaptation and de-adaptation are dependent on the training mechanism and intrinsic factors such as the participant's sensorimotor abilities. This study investigated the facilitation of the motor adaptation and de-adaptation processes for spatiotemporal features of an asymmetric gait pattern by sequentially applying split-belt treadmill (SBT) and asymmetric rhythmic auditory cueing (ARAC).

**Methods:**

Two sessions tested the individual gait characteristics of SBT and ARAC, and the remaining four sessions consisted of applying the two interventions sequentially during training. The adjustment process to the second intervention is referred to as “re-adaptation” and is driven by feedback error associated with the second intervention.

**Results:**

Ten healthy individuals participated in the randomized six-session trial. Spatiotemporal asymmetries during the adaptation and post-adaptation (when intervention is removed) stages were fitted into a two-component exponential model that reflects the explicit and implicit adaptation processes. A double component was shown to fit better than a single-component model. The decay constants of the model were indicative of the corresponding timescales and compared between trials. Results revealed that the explicit (fast) component of adaptation to ARAC was reduced for step length and step time when applied after SBT. Contrarily, the explicit component of adaptation to SBT was increased when it was applied after ARAC for step length. Additionally, the implicit (slow) component of adaptation to SBT was inhibited when applied incongruently after ARAC for step time.

**Discussion:**

These outcomes show that the role of working motor memory as a translational tool between different gait interventions is dependent on (i) the adaptation mechanisms associated with the interventions, (ii) the targeted motor outcome of the interventions; the effects of factors (i) and (ii) are specific to the explicit and implicit components of the adaptation processes; these effects are unique to spatial and temporal gait characteristics.

## 1 Introduction

Many post-stroke gait rehabilitation interventions are aimed at specific gait parameters such as step length, step time, walking speed, and cadence. These training processes often fall short of addressing all aspects of their gait pattern, necessitating the use of a combination of therapies. One example of combination therapy involves simultaneously applying split-belt treadmill (SBT) training and asymmetric rhythmic auditory cueing (ARAC) to target several aspects of gait. Additive effects on multiple gait features during concurrent application of these two training mechanisms indicate the possibility of independent neural networks driving the adaptation process (1). However, subjects adapt to these interventions at significantly different rates. This study focuses on the ability of one intervention type to facilitate the adaptation process to another intervention that places similar (or opposite) locomotor demands on the lower limb in healthy individuals while engaging a different motor adaptation mechanism. It was theorized that the working motor memory generated from adaptation to one intervention would help increase the rate of adaptation to the other, even though they are adapted through distinct processes. The outcome of this study should help in assembling combined interventions for subjects with neuromotor impairments who exhibit slow adaptation to certain training mechanisms.

Motor recovery is often limited by numerous sensory, cognitive, and motor deficits (2). Therefore, the ability of individuals to adapt or learn must be considered when determining effective gait rehabilitation techniques. Motor learning processes are driven by a multitude of factors and are controlled by different aspects of the nervous system (3). These processes are dependent on the type of perturbation that drives them (i.e., the therapy), the associated neural substrate, and the cognitive load exerted by the corresponding stimulation on the individual.

This study focuses on two interventions that fall under the first two motor learning categories: SBT and ARAC. The interventions were chosen because they impose a similar interlimb asymmetry while engaging different motor adaptation mechanisms. Stepping to rhythmic auditory cues is an instructive form of adaptation that exerts a substantial cognitive burden on the subject (feedforward mechanism), whereas SBT training is a more autonomous form of training relying on proprioceptive abilities (feedback mechanism, sensorimotor adaptation). These processes are dynamic, and their individual effects on one's locomotor adaptation are dependent on several environmental, cognitive, and proprioceptive factors.

The effects of these two interventions were examined by looking at changes in the rate of de-adaptation (returning to the gait pattern before the perturbation) and re-adaptation (adapting to a second perturbation immediately after exposure to another perturbation) for two gait parameters: step length asymmetry and step time asymmetry. Re-adaptation occurred during a second intervention while de-adaptation occurred during post-adaptation. Post-adaptation is the experimental stage after perturbations are removed and subjects return to their “unperturbed” walking conditions (i.e., same speed treads and no auditory cues). Re-adaptation is driven by feedback errors associated with the second intervention, as well as “savings” from the first intervention. “Savings” is a learning phenomenon in which individuals exhibit a smaller initial error with an increased rate of re-adaptation after their initial exposure to the training (4). This concept is referred to as “locomotor savings” when related to changes in gait patterns.

Adaptation mechanisms are often modeled as having multiple components, distinguished by their timescales which tend to vary in magnitude (5). A previous study evaluated the adaptation mechanism by modeling the rate of change of center-of-pressure asymmetry under three split-belt treadmill training (SBT) conditions (6). The outcomes showed a significant correlation between the slow component of the adaptation rate during the initial SBT perturbation and the fast component of the “re-adaptation” rate, suggesting the prospect of “savings” in facilitating the locomotor adaptation process. This may be attributed to the capacity of motor memory and the individual's ability to retrieve the memory while transferring their gait pattern from one context to the next (i.e., “generalizability”) (7).

The savings from both interventions, SBT and ARAC, increase with repetition. This was demonstrated in a previous study on healthy subjects in which repeated exposure to early adaptative training of SBT and washout resulted in enhanced re-adaptation (8). Additionally, the effects of SBT and ARAC superimpose when used simultaneously, indicating the possibility of independent neural pathways and corresponding adaptation mechanisms (1). In other words, the adaptation processes (i.e., sensorimotor adaptation and instructional adaptation) occur concurrently albeit at different timescales.

In the current study, the re-adaptation/de-adaptation rate(s) for spatiotemporal asymmetries were obtained through computational modeling of gait parameters during the adaptation (with asymmetric perturbation) and post-adaptation (without perturbation) stages of the experiment. Previous studies that modeled motor adaptation tasks made use of either an exponential model or a power-law model where the adaptation or de-adaptation indices were modeled as a function of time (9). These models are composed of one process (10, 11) or a combination of simultaneously occurring processes with different timescales (12, 13). A recent study characterized the overall motor adaptation process as consisting of at least two simultaneous processes that are categorized primarily by their distinct timescales (14). One of the adaptation mechanisms tends to be more fast-acting (explicit) and is understood to be lost just as rapidly (5). In contrast, the slower process (implicit) adapts to the perturbation at a moderate rate, but the adapted movement is lost relatively slowly as well (15). The explicit adaptation process generally involves more awareness and cognition than the implicit process; however, these correlations vary with different interventions (16).

In our study, SBT and ARAC are sequentially applied to test the effect of context on the “re-adaptation” rate, or “savings” (4). It was hypothesized that the spatiotemporal “savings” obtained via sensorimotor adaptation (i.e., SBT) would facilitate the rate of adaptation using “instructional” techniques (i.e., ARAC), and vice versa. Details of the hypotheses are outlined as follows:

(i) We hypothesized that an incongruent sequence would be less effective than a congruent sequence in enhancing the rate of “re-adaptation.” The similarity in assigned asymmetry between interventions in the congruent trials was expected to lead to a facilitated adaptation process. On the other hand, the incongruent sequence(s) imposed conflicting asymmetries between the interventions, that may have led to a potential inhibition in the adaptation process (17).
(ii) We hypothesized that the rate of change of interlimb asymmetry (measured by step time and step length) would be increased during “re-adaptation” because of working motor memory generalizing from one adaptation context to another when the two contexts impose equivalent gait asymmetry (congruent sequence). The compatibility in imposed asymmetry between the interventions aimed to facilitate re-adaptation, albeit via a different mechanism (17).

In addition, this study also compared the rate(s) at which acquired gait asymmetry was lost from sequential combinations of SBT and ARAC when the asymmetric perturbation was removed, i.e., the “de-adaptation” rate. This helps elucidate the interplay of different adaptation mechanisms as they pertain to the rate(s) of “re-adaptation” and “de-adaptation” upon removal of intervention.

## 2 Materials and methods

### 2.1 Participants

Seventeen healthy subjects were recruited within the University of South Florida – Tampa campus. The design of the experiment was approved by the University of South Florida Institutional Review Board (Pro #00016724). On their first visit, subjects were informed about the purpose of the study, the duration of each experiment, and the number of visits required. Upon their agreement, written informed consent was obtained from the individual. Subjects were required to meet the following eligibility criteria on their first visit, verified through an interview:

• No known neurological or gait impairments (self-reported).
• Minimum comfortable treadmill gait velocity of 1.0 m/s (assessed on first visit).
• No known injury or surgery in the past 24 months (self-reported).
• No known balance or proprioceptive issues (self-reported).
• No participation in any physical activity that requires a disproportionate use of one leg more than the other, e.g., soccer or skateboarding (self-reported).

All subsequent experiments were performed in accordance with the signed agreement. Subjects completed the Waterloo Footedness Questionnaire–Revised (WFQ-R) form, with the dominant leg determined by their response to the question about kicking a ball (18, 19). Participants were then invited to a baseline assessment, in which they were instructed to walk on a tied-belt treadmill for 3 min. The speed was increased from 0.9 m/s in increments equal to 0.1 m/s or smaller until confirmed by the subject that it was their comfortable walking speed. This speed was used for the baseline assessment to ensure eligibility and to determine comfortable walking speed and stride time. Motion capture and force data from this session were used to assess their baseline gait asymmetry. Individuals were only asked to return for the remaining sessions if their step length asymmetry and step time asymmetry were within the range of ±6% symmetry index (20) during this initial baseline assessment (21–23). Baseline values of their comfortable gait velocity and stride times obtained from this assessment were used to personalize all subsequent experiments.

### 2.2 Experiment design

**Figure 1:**
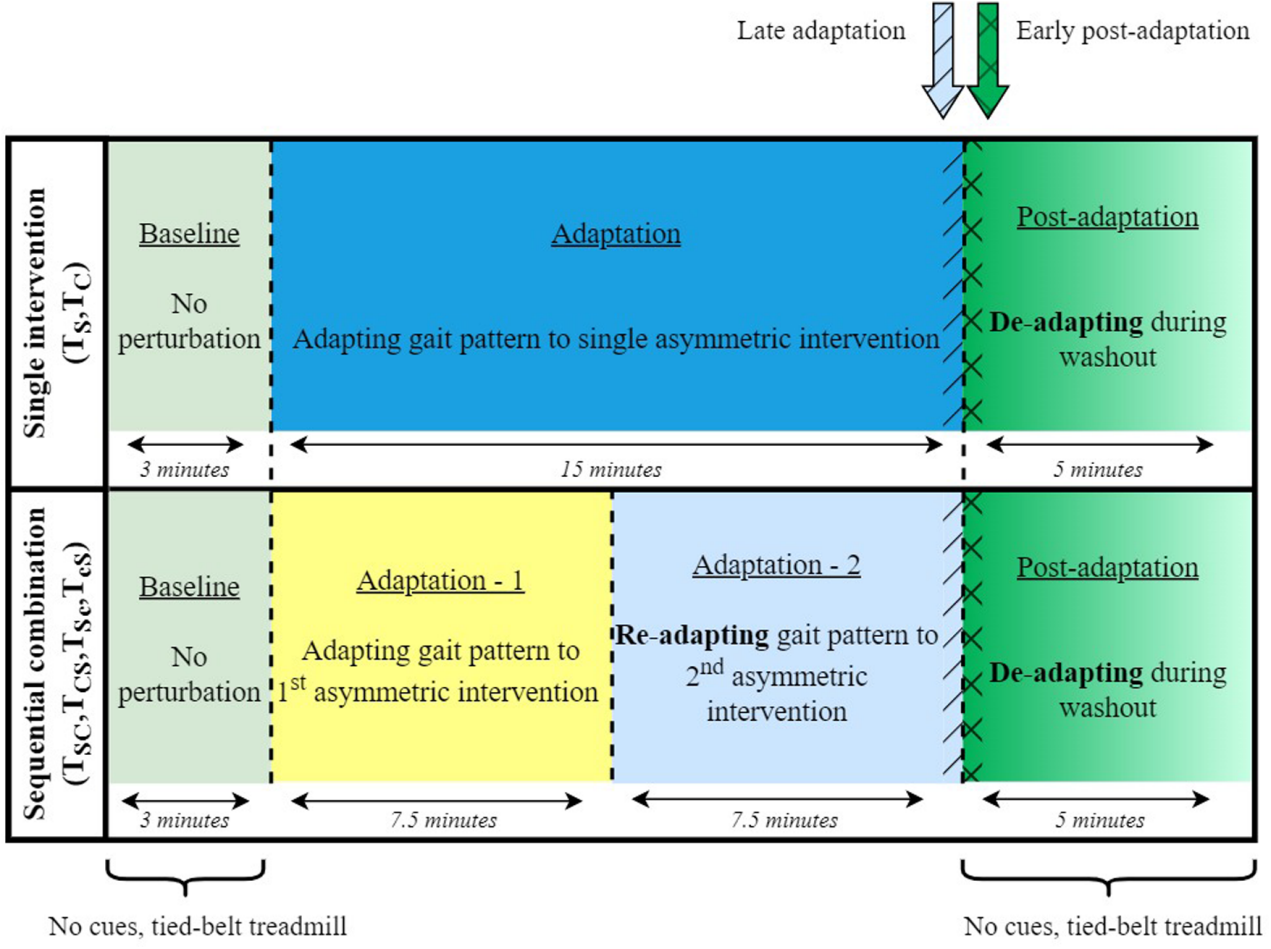
Experimental stages for single-intervention and sequentially combined trials. Each experiment involved 23 min of uninterrupted walking, including 15 min of training. Baseline and post-adaptation stages did not involve rhythmic cues, and lasted for 3 and 5 min, respectively. Average asymmetry during late adaptation was obtained over 15 strides before the end of adaptation (demonstrated in blue hatch patterns), and average asymmetry during early post-adaptation was obtained over 15 strides after the beginning of the post-adaptation stage (demonstrated in green crosshatch pattern).

**Figure 2:**
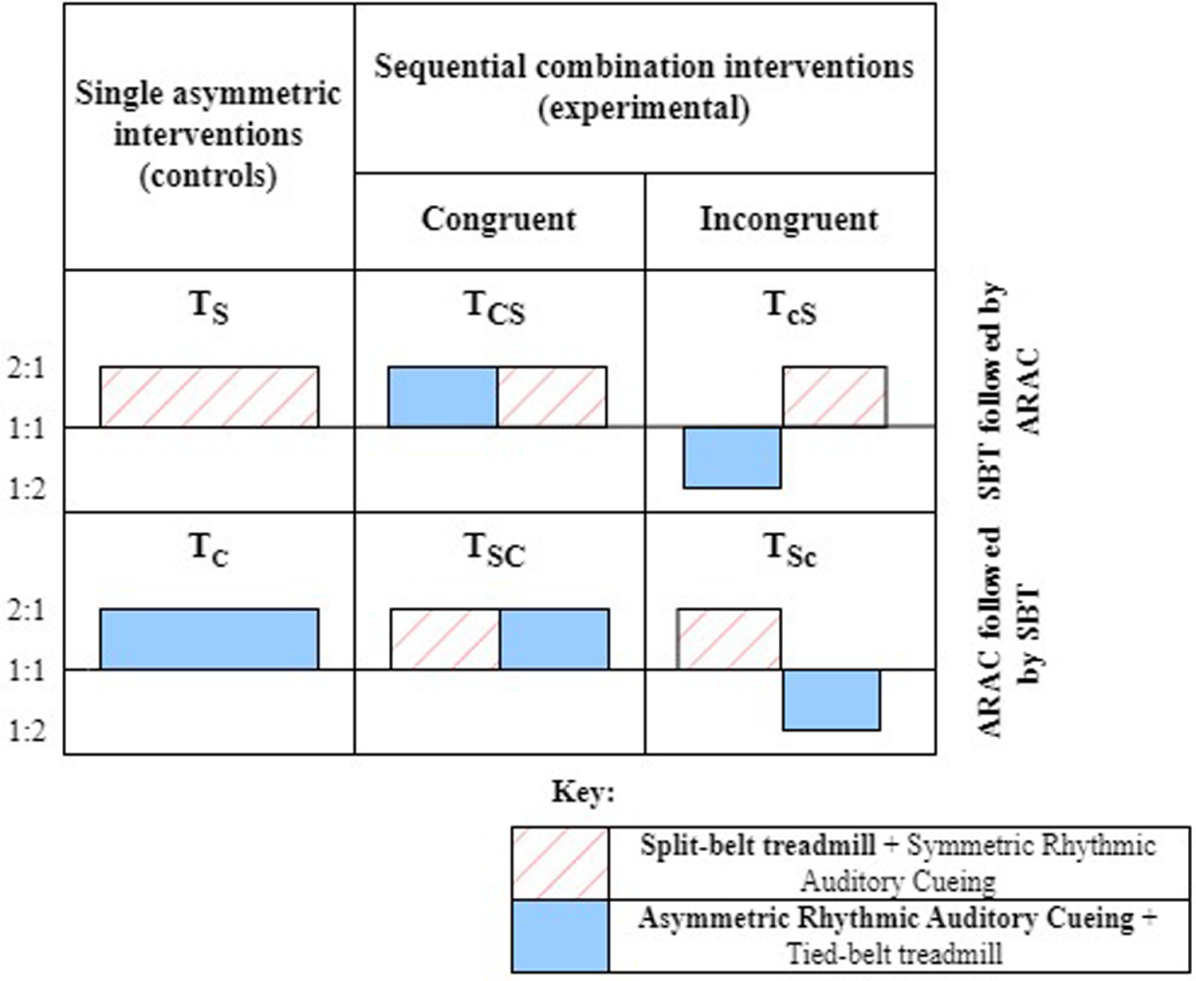
Trial conditions for individual and combined interventions during adaptation.

The study comprised six sessions, two of which involved single asymmetric interventions, and the remaining four sessions were sequential combinations of the two interventions. Out of the six sessions, two sessions involved evaluating the individual effect of each intervention technique (i.e., control trials) on the adaptation and de-adaptation rates $\left(T_{S},\;T_{C}\right)$. The remaining four sessions consisted of SBT and ARAC applied sequentially over the 15-minute adaptation stage so that each form of intervention was applied for 7.5 min during the adaptation period (Figure 1). The order of the sequence was reversed, along with its congruence so that ARAC applied the same magnitude of asymmetry as SBT in the ipsi- or contra-lateral limb. In other words, the congruent combinations consisted of sequentially applying SBT and ARAC so that the same direction of asymmetry would be applied by both interventions on the same lower limbs, whereas the incongruent combination consisted of the interventions applying asymmetries in opposite directions such that the effects would partially cancel out. The six experimental sessions are described in greater detail in Figure 2.

Two forms of asymmetric gait intervention techniques were used in this experiment: SBT and ARAC. For this study, symmetric rhythmic auditory cues were also played during SBT adaptation to ensure consistency in sensory load between SBT and ARAC. In SBT (2:1), the left belt speed was adjusted twice that of the right belt speed, while maintaining the individual's average gait velocity (Equations 1-1, 1-2). Symmetric rhythmic auditory cues were used during SBT adaptation to maintain consistent sensory load between SBT and ARAC training on a treadmill.

(1-1) $$\mathrm{Left}\;\;\mathrm{belt}\;\;\mathrm{speed}=\frac{4}{3}*\;\mathrm{comfortable}\;\mathrm{speed}\;$$

(1-2) $$\mathrm{Right}\;\mathrm{belt}\;\mathrm{speed}=\frac{2}{3}*\;\mathrm{comfortable}\;\mathrm{speed}\;$$

In ARAC (2:1), the interval between cues was adjusted so that the left step time was twice that of the right step time (Equations 2-1, 2-2) while maintaining the individual's comfortable stride time. The incongruent combination trials consisted of ARAC (1:2) where the left cue length of ARAC (2:1) is the same as the right cue length of ARAC (1:2), and vice versa for the contralateral limb.

(2-1) $$\mathrm{Left}\;\mathrm{cue}\;\mathrm{duration}=\frac{2}{3}*\;\mathrm{comfortable}\;\mathrm{stride}\;\mathrm{time}$$

(2-2) $$\;\mathrm{Right}\;\mathrm{cue}\;\mathrm{duration}=\frac{1}{3}*\;\mathrm{comfortable}\;\mathrm{stride}\;\mathrm{time}$$

The order in which subjects performed the trials was randomized to minimize after-effects from previous sessions. Randomization was performed using a Latin square, resulting in six distinct trial orders, as shown in Table 1. These trial orders were evenly distributed among the subject population for randomization consistency, and consecutive sessions were scheduled a minimum of 24 hours apart.

**Table 1:** Randomization of trial order.

Trial order	Sequence of trials
I	$T_{S}\to T_{C}\to T_{cS}\to T_{SC}\to T_{Sc}\to T_{CS}$
II	$T_{C}\to T_{SC}\to T_{S}\to T_{CS}\to T_{cS}\to T_{Sc}$
III	$T_{SC}\to T_{CS}\to T_{C}\to T_{Sc}\to T_{S}\to T_{cS}$
IV	$T_{CS}\to T_{Sc}\to T_{SC}\to T_{cS}\to T_{C}\to T_{S}$
V	$T_{Sc}\to T_{cS}\to T_{CS}\to T_{S}\to T_{SC}\to T_{C}$
VI	$T_{cS}\to T_{S}\to T_{Sc}\to T_{C}\to T_{CS}\to T_{SC}$

### 2.3 Experimental apparatus

**Figure 3:**
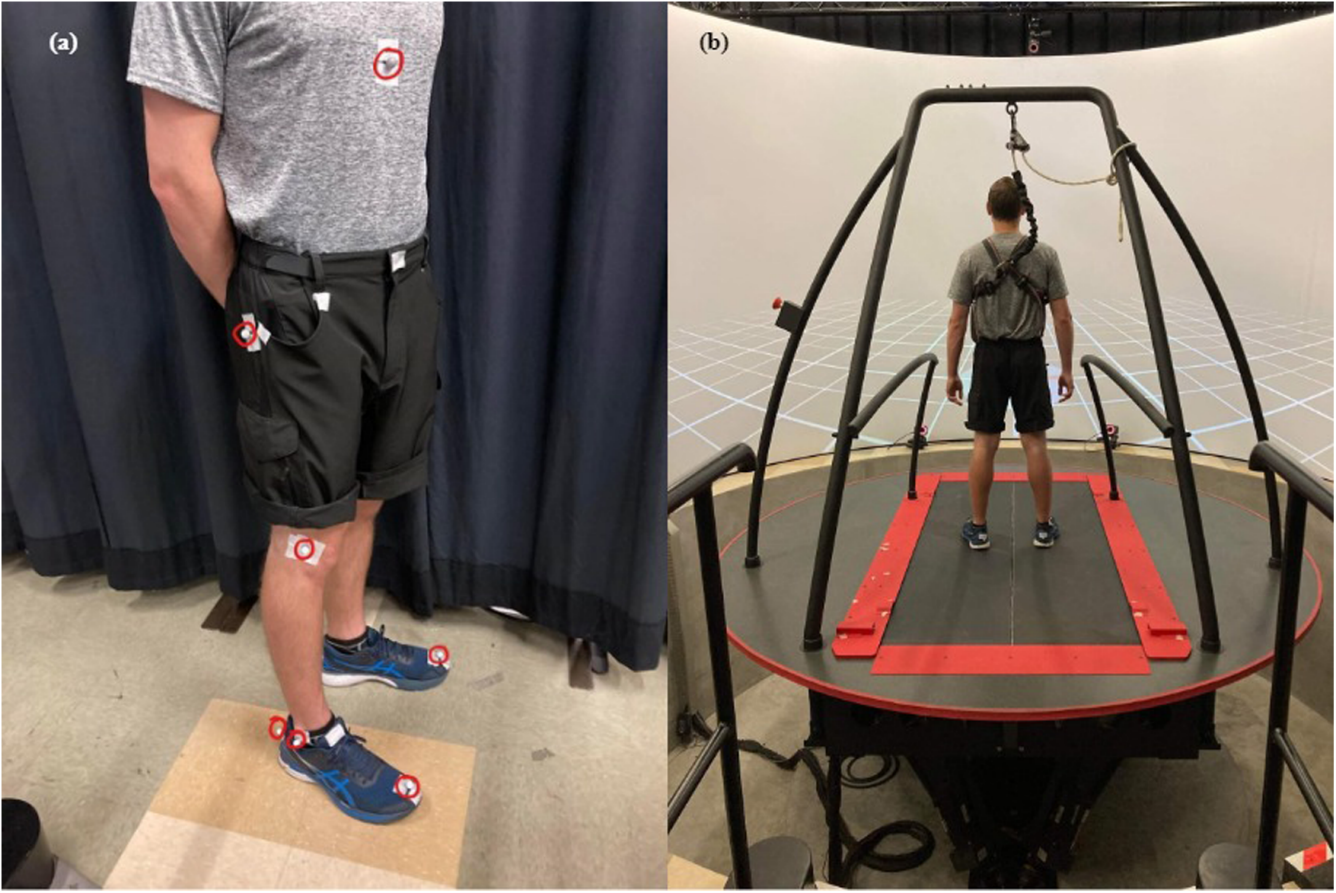
Subject **(a)** with eleven infrared reflective markers circled in red and **(b)** on the CAREN (Computer-Assisted Rehabilitation Environment) platform at the beginning of an experiment.

Experiments were performed on the CAREN (Computer Assisted Rehabilitation Environment, Motek Medical), which includes a platform with six degrees of freedom equipped with a split-belt treadmill, 180-degree projection screen, and surround sound for auditory cueing. The platform was also equipped with force plates and surrounded by 10 Vicon infrared cameras for motion capture. D-Flow, a visual programming tool, was used to develop the protocol for the baseline assessment and the six experimental trials. It was also used to collect time-series of motion capture and force plate data. During the experiments, eleven infrared reflective markers were placed on the subject's lower limb joints for motion capture. Markers were placed on the subject's big toes, heels, lateral malleoli, menisci of knee joints, tips of greater trochanters, and the bottom of the sternum (Figure 3a). Subjects were also fitted with a safety harness before the start of each trial. The harness length was adjusted to ensure the safety of the individual and then attached to the guard rail on top of the platform (Figure 3b). Data was collected at a frequency of 100 Hz using the Vicon motion capture system. Subsequent data processing, gait analysis, and motor modeling were performed using MATLAB 2021a, and statistical analyses were performed on IBM SPSS Statistics 28.

### 2.4 Data analysis

Marker trajectory data were processed using MATLAB 2021a. Heel positions and force data were used to determine the step length and step times throughout the experiment. This was used to obtain gait asymmetries for step length and step time using Equation 3, also referred to as the symmetry index (20). A negative value obtained for “% Asymmetry” indicates that the corresponding gait parameter is greater in magnitude on the right leg compared to that on the left leg.

(3) $$\text{\%}\;\mathrm{Asymmetry}=\frac{\mathrm{Left}\;\mathrm{step}-\mathrm{Right}\;\mathrm{step}}{\mathrm{mean}(\mathrm{Left}\;\;\mathrm{step},\;\;\;\mathrm{Right}\;\;\mathrm{step})}*100$$

Obtained asymmetries were then passed through a 1st order Butterworth filter with a cutoff frequency of 50 Hz. The time course of gait asymmetry acquired from SBT training has been previously studied (10, 11, 13, 24) and modeled using single-component or double-component exponential models (Equations 4-1, 4-2).

(4-1) $$\;\text{\%}\;{\mathrm{Asymmetry}}_{\mathrm{single}}(t)=(a*e^{-b(t)})+c$$

(4-2) $$\;\text{\%}\;{\mathrm{Asymmetry}}_{\mathrm{double}}(t)=(a*e^{-b(t)})+(d*e^{-f(t)})+c$$

The decay constants obtained from the exponential model(s), i.e., term “*b*” from Equation 4-1, and terms “*b*,” and “*f*” from Equation 4-2, are indicative of the rate at which gait asymmetry is acquired during adaptation and also represents the rate at which the acquired gait asymmetry was lost during post-adaptation; the components “a” and “d” indicate the amplitude of the exponential functions, i.e., they represent the initial component (at time = 0 sec) of the corresponding decay process(es). The control trials $\left(T_{S}\;\mathrm{and}\;T_{C}\right)$ had an adaptation period of 15 min, which was greater than the adaptation period for the sequential combination trials. To ensure consistency of steady-state values between the control and experimental conditions for the non-linear regression models, asymmetries were obtained from strides within the first 7.5 min of the adaptation period for the control trials, $T_{S}$, and $T_{C}$.

**Figure 4:**
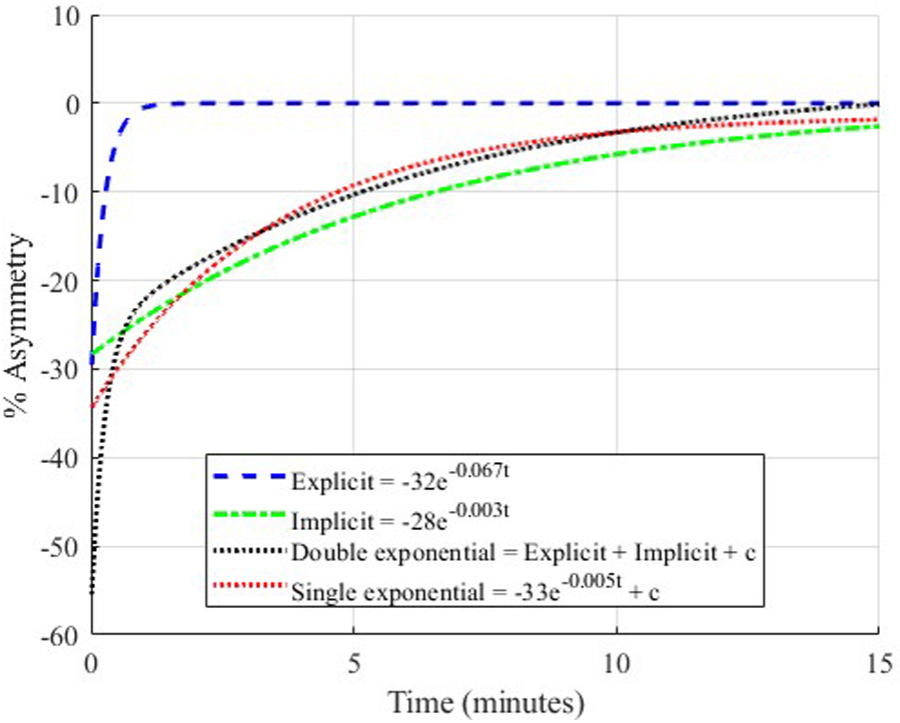
Demonstration of single-component and double-component exponential models. The two exponentials that make up the double-component model are shown individually as explicit (the fast process) and implicit (the slow process).

Gait asymmetry over time was obtained using Equation 3, and then a non-linear technique known as Particle Swarm Optimization (PSO) was used to minimize any tradeoff between error and cost, resulting in modulated parametric bounds for the non-linear model (25). Error is the discrepancy between the actual and modeled values, whereas the cost is a measure of the solution based on the fitness model. PSO utilizes a mechanism in which the local velocity of each data point in random directions is assessed and compared against the global minimum of the function with each iteration. Optimization was performed twice on the raw data before curve-fitting to select the model (single or double exponential) with the lower cost. The outputs (i.e., coefficients, decay constants, and steady-state constant) were found once convergence (i.e., additional iterations do not improve the fitted model) was established between the output and the raw data. The algorithm used for optimization and curve-fitting was performed using Rashid et al. 2020's algorithm on MATLAB 2021a (26). Data were fitted to both models for comparison of goodness-of-fit because the literature is inconclusive about whether the (de)adaptation process should be modelled as a single-component or double-component exponential. Single-component and double-component exponential functions were fitted to the optimized data for every individual; an example is shown in Figure 4.

Residual distribution was checked for normality in their distribution for both model types using histograms and QQ plots. Upon determination of residual distribution normality, a quantitative comparison was made to determine the goodness-of-fit of the two exponential models using Akaike's Information Criterion (AIC). AIC adds a penalty term to compensate for the complexity of the model and is a useful tool for evaluating the goodness-of-fit for nonlinear regression models (27). Lower indicates better, so the model that resulted in an AIC closer to negative infinity was chosen as the better fit between the exponential models.

The exponential models generated represented the step length asymmetry and step time asymmetry over the adaptive and post-adaptive stages of the experiments. Decay constant(s) generated from the fitted models were indicative of the rate of adaptation and de-adaptation. The decay constants exhibited the progression of the explicit and implicit components of the adaptation process. The following comparisons were made for corresponding decay rates to address the study objectives:

• Between $T_{S}$, $T_{CS}$, and $T_{cS}$ during adaptation-2 to determine the effects of ARAC on (i) facilitating (or inhibiting) motor adaptation to SBT, and (ii) de-adaptation rate from SBT.
• Between $T_{C}$, $T_{SC}$, and $T_{Sc}$ during adaptation-2 to determine the effects of SBT on facilitating (or inhibiting) motor adaptation to ARAC, and (ii) de-adaptation rate from ARAC.
• Between $T_{S}$ and $T_{C}$ to determine differences in the rate of adaptation/de-adaptation pertaining to the primary interventions.

It must be noted that adaptation of one's gait to ARAC requires active compliance on the subject's end for the intervention to result in significant changes in their gait pattern. The effectiveness of rhythmic cueing has been known to be closely associated with the subjects’ rhythm production abilities (28). Since compliance is dynamic and may change throughout the study, this might have limiting effects on the outcome interpretability.

All datasets were checked for normality violation using the Shapiro-Wilk test before statistical analysis. For cases where normality was violated (*p* < 0.05), non-parametric statistical methods – Friedman's test was used instead of repeated measures analysis of variance for statistical assessment. *Post hoc* pairwise comparisons were performed using the Wilcoxon signed-rank test for non-parametric tests.

## 3 Results

### 3.1 Subjects

Seventeen subjects were invited to participate in the study. Four subjects (S-1, S-2, S-12, S-17) did not meet the inclusion criteria based on their baseline gait assessment, and two subjects (S-5, S-7) chose to discontinue their participation after their first session. This resulted in a total of eleven eligible participants who completed all six sessions of the study. The subjects’ demographics and other relevant information are outlined in Table 2.

**Table 2:** Subject information.

Subject	Trial order	Gender	Dominant leg	Baseline gait velocity (m/s)	Baseline step length asymmetry (%)	Baseline step time asymmetry (%)
S-3	III	M	L	1.15	2.4	5.2
S-4	IV	M	R	1.15	2.5	2.1
S-6	VI	M	R	1.10	−0.5	−2.9
S-8	II	F	R	1.00	−1.1	4.7
S-9	III	F	R	1.00	−2.3	−1.8
S-10	IV	M	R	1.17	−1.4	2.0
S-11*	V	F	R	1.10	3.3	−3.8
S-13	I	M	R	1.20	−5.0	−2
S-14	V	F	L	1.15	1.2	2.6
S-15	VI	F	L	1.00	1.1	−1.6
S-16	I	M	R	1.10	1.1	0.9

The table outlines the eleven eligible subjects’ assigned randomized trial order, dominant leg, step length asymmetry (0.12 ± 2.45%), and step time asymmetry (0.49 ± 3.08%) at baseline conditions on the day of initial eligibility assessment. A negative asymmetry indicates that the gait parameter was relatively greater in magnitude on the right limb.

* Subject 11 was not included in the study due to a very high baseline during trial 5.

S-11 returned on her fifth trial (i.e., $T_{SC}$) with an exceptionally high baseline step length asymmetry, −11.8%. This asymmetry exceeded the allowable range of step length asymmetry and was also twice the value of the second highest baseline step length asymmetry. Consequently, S-11 was removed from all subsequent analyses to limit the effect of this inconsistency on the outcomes. The resulting subject population (*n* = 10) included two subjects whose characteristics may have affected the overall average: S-6's spatiotemporal gait asymmetry was increased upon removal of perturbation during $T_{C}$ (Figure in Supplementary Material) and S-14 had a misaligned femur-patella, resulting in a leg length discrepancy (LLD). Her left leg length was greater than that of the right.

### 3.2 Modeling outcomes

The model assessment was performed by comparing the AIC for the single-component and double-component exponential models for step length asymmetry and step time asymmetry during adaptation and post-adaptation stages for the control and combination trials. AIC for the control trials, $T_{S}$ and $T_{C}$, are shown in Tables 3 and 4. Similarly, the models depicting the process of re-adaptation and de-adaptation of step length asymmetry were better represented by the double-component exponential model in the sequential interventions, except the de-adaptation process of $T_{Sc}$. These results corroborated Rashid et al. 2020's outcome by modeling the adaptation and post-adaptation stages of step length asymmetry under split-belt treadmill training conditions and showed that a similar computational model is a better fit for another intervention technique, ARAC (13). AIC for the sequential combination trials during adaptation and post-adaptation stages can be found under Supplementary Material.

**Table 3:** Akaike information criterion (AIC) of step length asymmetry for single-component and double-component exponential models during adaptation and post-adaptation of $T_{S}$ and $T_{C}$.

Exponential model type	$T_{S}$		$T_{C}$	
	Adaptation	Post-adaptation	Adaptation	Post-adaptation
Single-component	−648.87	−5,328.33	−6,650.17	−7,441.56
Double-component	−2,414.12	−5,585.47	−6,960.22	−7,470.11

**Table 4:** Akaike information criterion (AIC) of step time asymmetry for single-component and double-component exponential models during adaptation and post-adaptation of $T_{S}$ and $T_{C}$.

Exponential model type	$T_{S}$		$T_{C}$	
	Adaptation	Post-adaptation	Adaptation	Post-adaptation
Single-component	−1,858.62	−5,989.10	−3,705.22	−7,396.54
Double-component	−2,313.33	−6,028.45	−3,799.18	−7,416.53

The residual distribution histogram and QQ plots showed normality for all trials during adaptation and post-adaptation. Figures showing their distribution can be found under Supplementary Material. AIC was used as a quantitative measure to determine the best-fit model between the two non-linear regression models. A lower value (closer to negative infinity is better) of AIC was obtained for the double-component exponential models for both gait parameters during the adaptation and de-adaptation processes. Since the double-component model exhibited a lower AIC value than the single-component model for both trials, it was determined to be a better modeling fit for the adaptation and de-adaptation processes (Tables 3, 4).

In addition, the double-component exponential model was a better fit for step time asymmetry for all regression models pertaining to adaptation and de-adaptation. Since the double-component exponential model was a better fit for modeling adaptation/de-adaptation of step time asymmetry in all contexts, it was used for succeeding analyses.

The double-component exponential models generated two decay constants that are indicative of the adaptation/de-adaptation process. The implicit process was the exponent with a lower value component of the obtained decay constants (indicating slower changes), and the explicit process was the exponents with a higher value time constant (indicating faster changes).

### 3.3 Step length

The Shapiro-Wilk test revealed that the distribution of seven explicit components (out of twelve) and nine implicit components (out of twelve) were non-normal, as shown in Table 5. Since most of them were non-normal, non-parametric methods were chosen for statistical analyses. Additionally, the distribution of the decay constants was displayed using a box-and-whisker plot, discussed below.

**Table 5:** Shapiro-Wilk test results showing normality in distribution of decay constants pertaining to step length asymmetry during adaptation and post-adaptation of the six trials.

Experiment stage		$T_{S}$	$T_{C}$	$T_{SC}$	$T_{CS}$	$T_{Sc}$	$T_{cS}$
Explicit	Adaptation	*W* = 0.879, *p* = 0.128	*W* = 0.854, *p* = 0.064	*W* = 0.556, *p* < 0.001	*W* = 0.784, *p* = 0.009	*W* = 0.768, *p* = 0.006	*W* = 0.753, *p* = 0.004
	Post-adaptation	*W* = 0.859, *p* = 0.074	*W* = 0.858, *p* = 0.073	*W* = 0.512, *p* < 0.001	*W* = 0.580, *p* < 0.001	*W* = 0.851, *p* = 0.059	*W* = 0.574, *p* < 0.001
Implicit	Adaptation	*W* = 0.803, *p* = 0.016	*W* = 0.572, *p* < 0.001	*W* = 0.902, *p* = 0.230	*W* = 0.551, *p* < 0.001	*W* = 0.889, *p* = 0.164	*W* = 0.515, *p* < 0.001
	Post-adaptation	*W* = 0.895, *p* = 0.193	*W* = 0.724, *p* = 0.002	*W* = 0.523, *p* < 0.001	*W* = 0.501, *p* < 0.001	*W* = 0.547, *p* < 0.001	*W* = 0.410, *p* < 0.001

The normality results for the decay constant distribution in the sequential combination trials for the adaptation period correspond only to adaptation-2 of those experiments.

**Figure 5:**
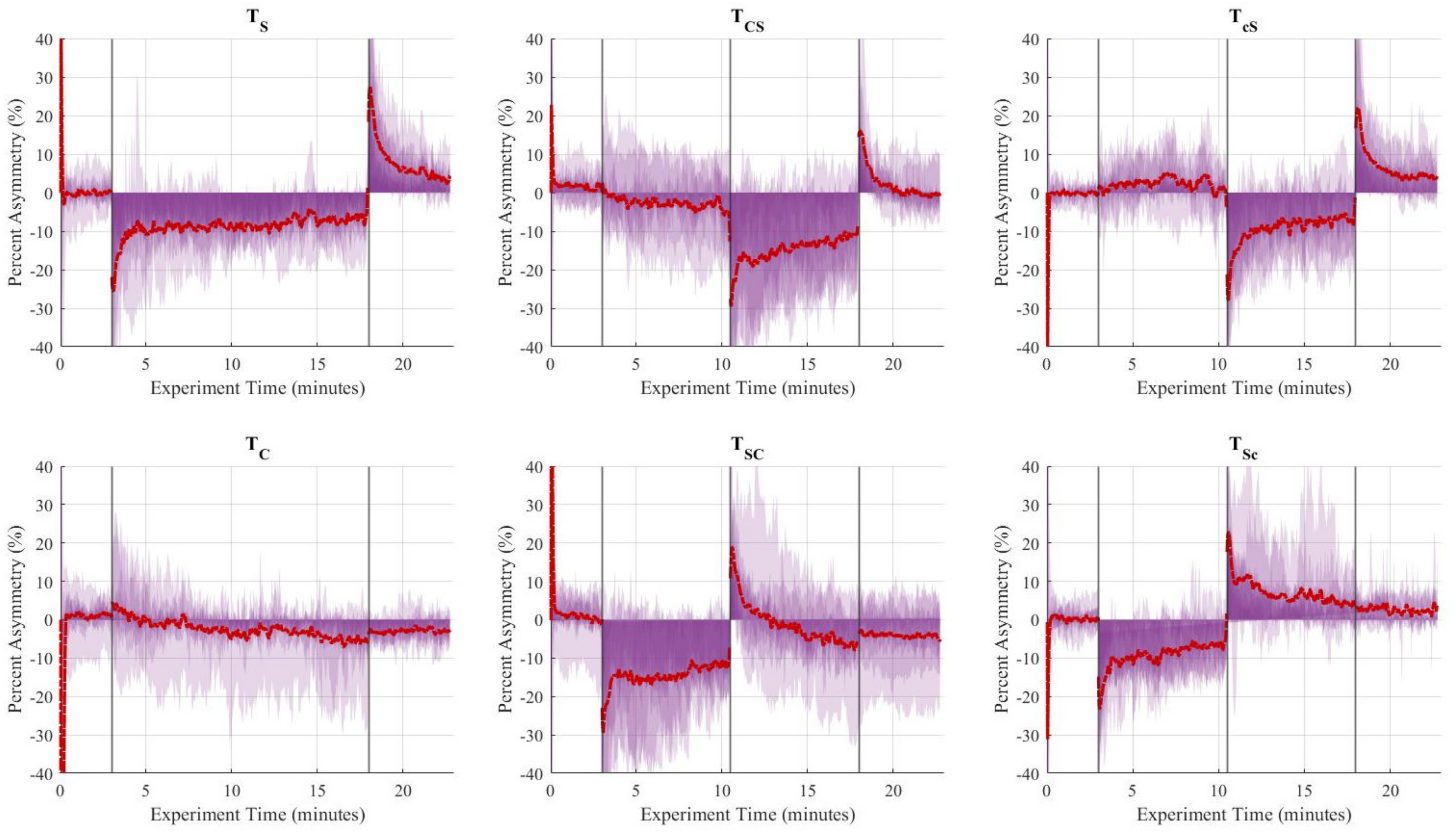
Aggregate (*n* = 10) step length asymmetry (%) over time for the six trials. The vertical lines separate the experimental stages: baseline (3 min), adaptation-1 (7.5 min), adaptation-2 (7.5 min), and post-adaptation (5 min). The single asymmetric interventions $\left(T_{S},T_{C}\right)$ were applied continuously during the 15-minute adaptation period. The red dashed line represents the group's average asymmetry throughout the experiments, and the translucent purple areas are indicative of the gait asymmetry of each individual throughout the experiment.

Friedman's test revealed that the difference in step length asymmetry was statistically significant (*p* < 0.05) between baseline, late adaptation, and early post-adaption stages for all trial types except sequential combinations in which SBT was first and ARAC was second, i.e., $T_{SC}$ and $T_{Sc}$ (Table 6). Step length asymmetry of the subject population during the six trials is shown in Figure 5. Signed-rank tests revealed that the difference in step length asymmetry between (i) baseline and late adaptation, and (ii) baseline and early post-adaptation were statistically significant (*p* < 0.05) for $T_{S}$, $T_{C},\;T_{CS}$, and $T_{cS}$, as shown in Table 7.

**Table 6:** Friedman's test results showing statistical differences in step length asymmetry among baseline, late adaptation, and early post-adaptation for the six trial types.

	$T_{S}$ *	$T_{C}$ *	$T_{SC}$	$T_{CS}$ *	$T_{Sc}$	$T_{cS}$ *
$\chi^{2}(2)$	18.2	6.2	2.4	16.8	2.6	20.0
*p*-value	<0.001	0.045	0.301	<0.001	0.273	<0.001

* Statistical significance at the 0.05 level is shown with an asterisk.

**Table 7:** Wilcoxon signed-rank results for step length asymmetry between (i) baseline and late adaptation, and (ii) baseline and early post-adaptation.

	$T_{S}$**	$T_{C}$**	$T_{CS}$**	$T_{cS}$**
Baseline vs. Late adaptation	*Z* = −2.701, *p* = 0.007	*Z* = −2.191, *p* = 0.028	*Z* = −2.191, *p* = 0.028	*Z* = −2.803, *p* = 0.005
Baseline vs. Early post-adaptation	*Z* = 2.803, *p* = 0.005	*Z* = −2.293, *p* = 0.022	*Z* = −2.803, *p* = 0.005	*Z* = −2.803, *p* = 0.005

Statistical significance at the 0.05 level is indicated with two asterisks (**) representing significant differences between (i) baseline and late adaptation, and (ii) baseline and early post-adaptation.

#### 3.3.1 Facilitation of adaptation to SBT and ARAC for step length asymmetry

**Figure 6:**
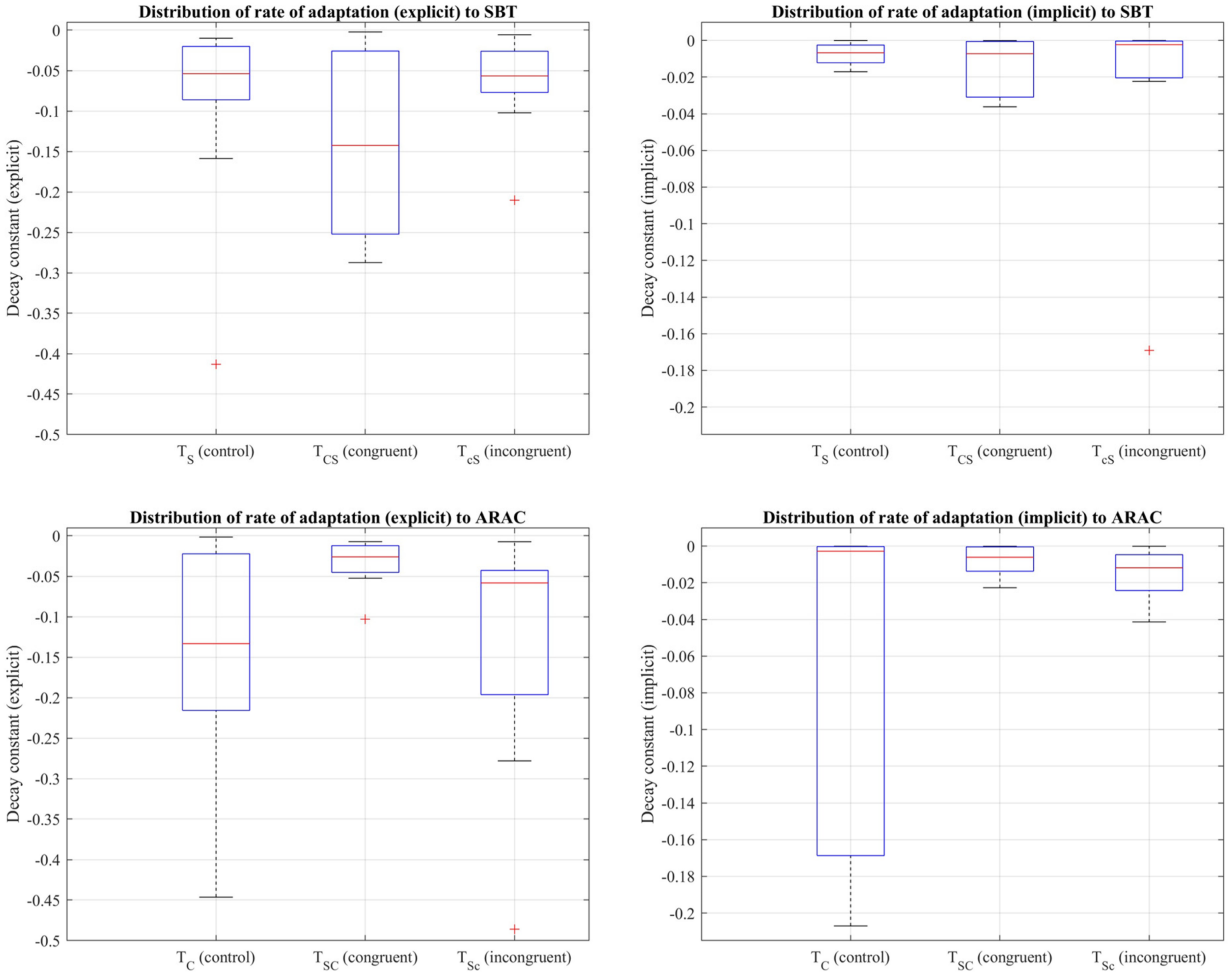
Distribution of decay constants depicting the explicit (left column) and implicit (right column) components of the adaptation rate of step length asymmetry (%) to SBT (top row) and ARAC (bottom row) during the first half of the adaptation period of $T_{S}$, $T_{C}$, and adaptation-2 for the sequential combination trials demonstrated using a box-and-whisker plot. The box outlined in blue shows the interquartile range, the red horizontal line indicates the median of the distribution, and the outliers are shown using the red “+” symbols.

Decay constants to quantify the rate of adaptation were obtained from the first 7.5 min of the adaptation period of the control trials $\left(T_{S},T_{C}\right)$ and over adaptation-2 of the sequential combination trials $\left(T_{SC},T_{CS},T_{Sc},T_{cS}\right)$. Friedman's test revealed that there was a statistically significant difference between the control trial and corresponding sequential combinations in the explicit component of the rate of adaptation to (i) SBT: $\chi^{2}(2)\;=\;7.2,\;p\;=\;0.027$, and (ii) ARAC: $\chi^{2}(2)\;=\;7.8,\;p\;=\;0.020$ (distributions shown in Figure 6). Results of multiple pairwise comparisons, in addition to the difference in outcome between the control trials, $T_{C}$ and $T_{S}$, are shown in Table 8.

**Table 8:** Wilcoxon signed-rank test results for the explicit component of the adaptation rate of step length asymmetry.

	$T_{C}-T_{S}$*	$T_{CS}-T_{S}$*	$T_{cS}-T_{S}$*	$T_{cS}-T_{CS}$	$T_{SC}-T_{C}$*	$T_{Sc}-T_{C}$	$T_{Sc}-T_{SC}$
*Z*	−2.191	−2.395	−2.191	−1.886	−2.090	−0.459	−1.886
*p*-value	0.028	0.017	0.028	0.059	0.037	0.646	0.059

The asterisk (*) indicates statistical significance (*p* < 0.05) in the difference between the explicit component associated with the second half of the adaptation process for the specified trial types.

Friedman's test also showed that the implicit rate of adaptation was not significantly different among $T_{S}$, $T_{CS}$, and $T_{cS}$ at a significance level of 0.05: $\chi^{2}(2)\;=\;2.6,\;p\;=\;0.2725$. Similar results were obtained for the implicit rate of adaptation to ARAC: $\chi^{2}(2)\;=\;0.6,\;p\;=\;0.7408$, indicating that the difference in the implicit decay constant among $T_{C}$, $T_{SC}$, and $T_{Sc}$ was not statistically significant.

#### 3.3.2 Rate of de-adaptation for step length asymmetry

**Figure 7:**
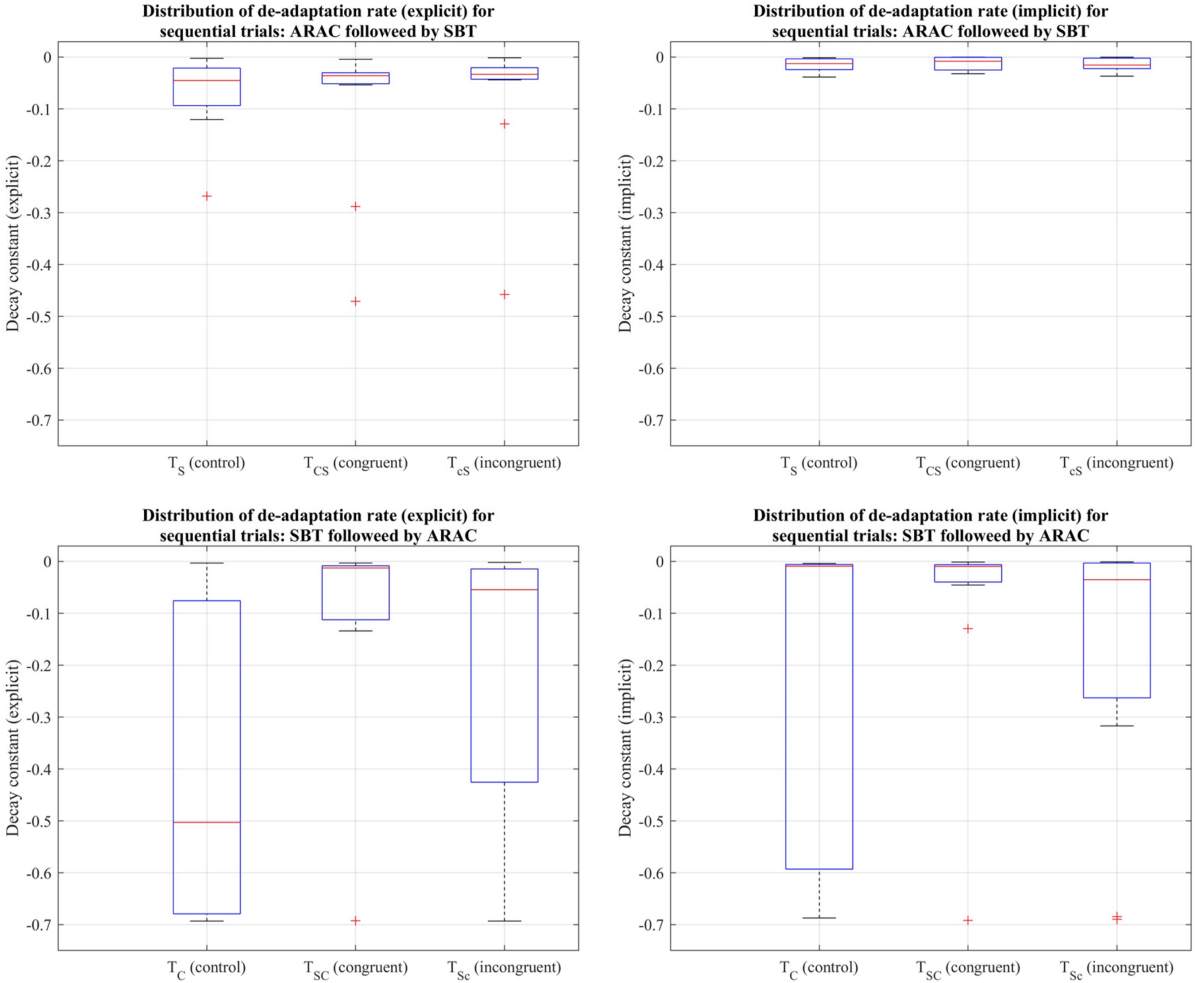
Distribution of decay constants depicting the explicit (left column) and implicit (right column) components of de-adaptation between the six trial types for step length asymmetry demonstrated using a box-and-whisker plot. The top row demonstrates distribution of de-adaptation rate from SBT and trials in which ARAC is followed by SBT; the bottom row demonstrates distribution of de-adaptation rate from ARAC and trials in which SBT is followed by ARAC. The box outlined in blue shows the interquartile range, the red horizontal line indicates the median of the distribution, and the outliers are shown using the red “+” symbols.

The decay constants depicting the explicit and implicit components in gait de-adaptation were compared among the six trial types during the post-adaptation period, where subjects returned to tied-belt treadmill walking at their comfortable speed. Friedman's test revealed that the differences among the six trial types were statistically insignificant (*p* > 0.05) for the (i) explicit process: $\chi^{2}(5)\;=\;9.49,\;p\;=\;0.0912$, and (ii) implicit process: $\chi^{2}(5)\;=\;4.91,\;p\;=\;0.4264$ (distributions shown in Figure 7).

### 3.4 Step time

**Figure 8:**
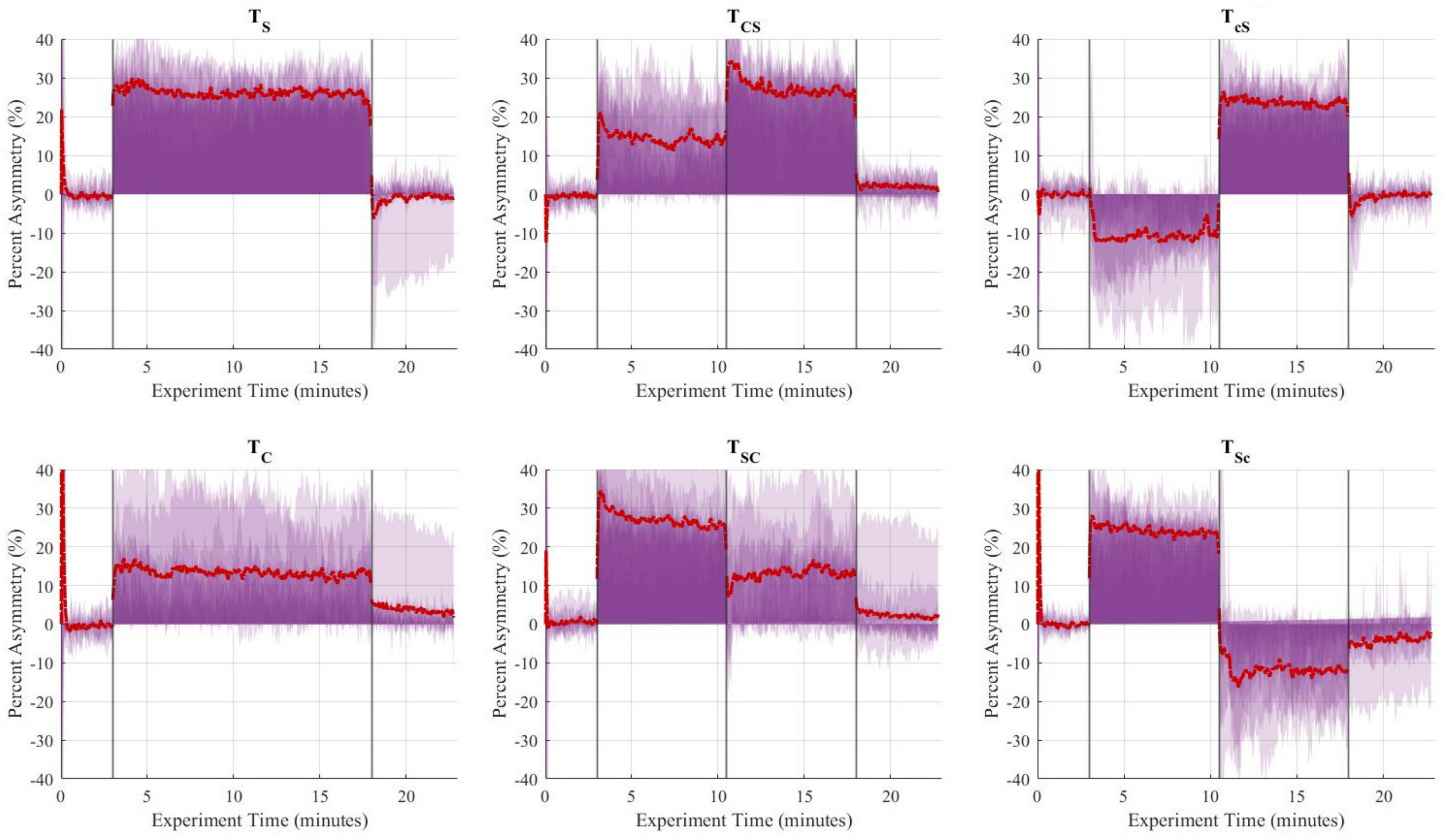
Aggregate (*n* = 10) step time asymmetry (%) over time for the six trials. The vertical lines separate the experimental stages: baseline (3 min), adaptation-1 (7.5 min), adaptation-2 (7.5 min), and post-adaptation (5 min). The single asymmetric interventions $\left(T_{S},T_{C}\right)$ were applied continuously during the 15-minute adaptation period. The red dashed line represents the group's average asymmetry throughout the experiments, and the translucent purple areas are indicative of the gait asymmetry of each individual throughout the experiment.

**Figure 9:**
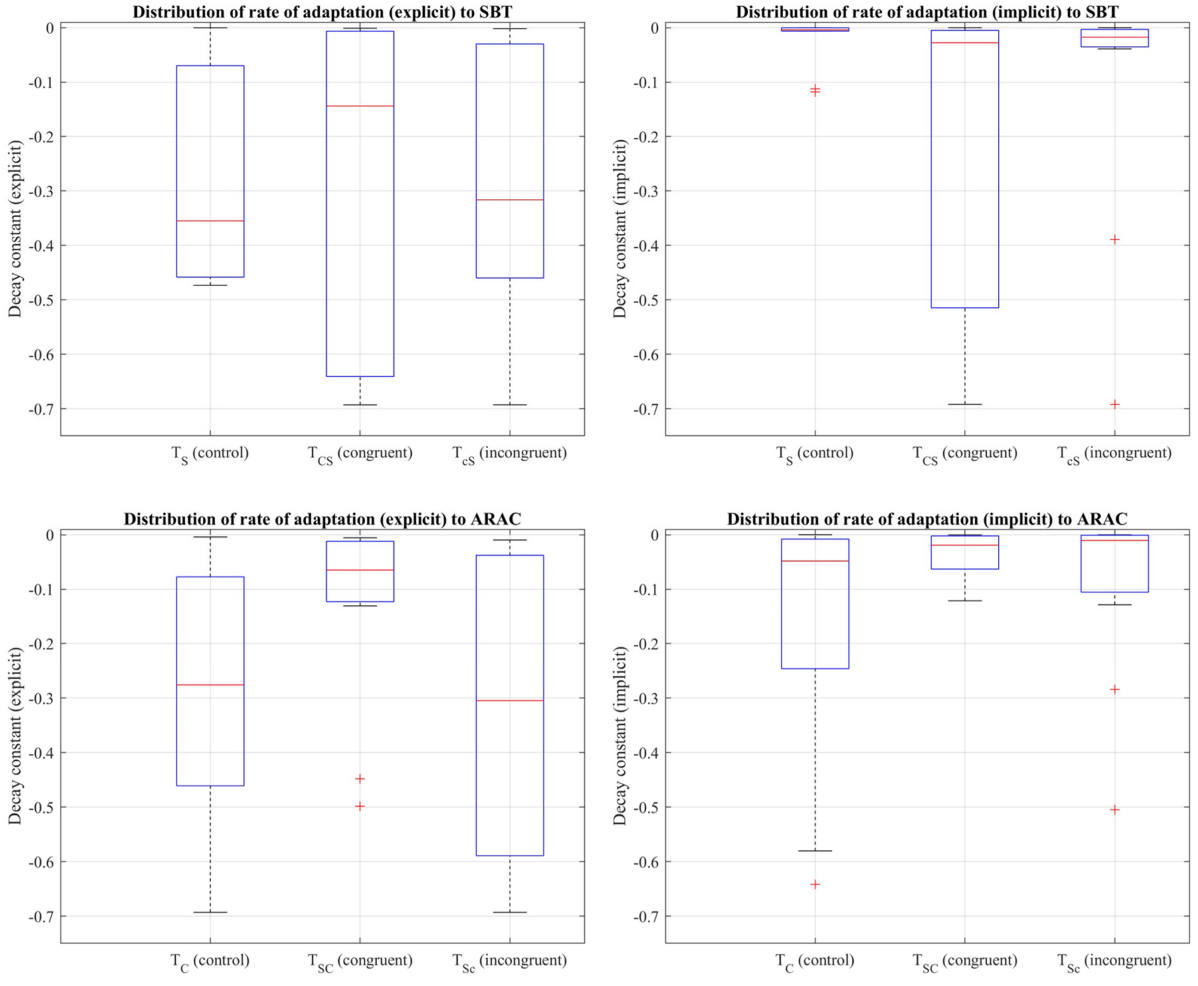
Distribution of decay constants depicting the explicit (left column) and implicit (right column) components of the adaptation rate of step time asymmetry (%) to SBT (top row) and ARAC (bottom row) during the first half of the adaptation period of $T_{S}$, $T_{C}$, and adaptation-2 for the sequential combination trials demonstrated using a box-and-whisker plot. The box outlined in blue shows the interquartile range, the red horizontal line indicates the median of the distribution, and the outliers are shown using the red “+” symbols.

The Shapiro-Wilk test revealed that the distribution of seven explicit components (out of twelve) were non-normal (i.e., rate of 3 adaptation and 4 de-adaptation processes) and all twelve implicit components were normal, as shown in Table 9. Similar to the statistical analyses for step length, the more robust non-parametric method (i.e., Friedman's test and pairwise comparisons using Wilcoxon sign-rank test) was chosen for step time. Figure 8 shows the step time asymmetry throughout the experiment for all six trial types. The distribution of decay constants for step time asymmetry was displayed using a box-and-whisker plot to accurately display the variability in asymmetry and skewness between different conditions (Figure 9).

**Table 9:** Shapiro-Wilk test results showing normality in distribution of decay constants pertaining to step length asymmetry during adaptation and post-adaptation of the six trials.

Experiment stage		$T_{S}$	$T_{C}$	$T_{SC}$	$T_{CS}$	$T_{Sc}$	$T_{cS}$
Explicit	Adaptation	*W* = 0.758, *p* = 0.004	*W* = 0.903, *p* = 0.238	*W* = 0.718, *p* = 0.001	*W* = 0.726, *p* = 0.002	*W* = 0.863, *p* = 0.082	*W* = 0.924, *p* = 0.392
	Post-adaptation	*W* = 0.927, *p* = 0.419	*W* = 0.769, *p* = 0.006	*W* = 0.828, *p* = 0.032	*W* = 0.943, *p* = 0.588	*W* = 0.795, *p* = 0.012	*W* = 0.841, *p* = 0.045
Implicit	Adaptation	*W* = 0.808, *p* = 0.018	*W* = 0.466, *p* < 0.001	*W* = 0.683, *p* < 0.001	*W* = 0.757, *p* = 0.004	*W* = 0.669, *p* < 0.001	*W* = 0.416, *p* < 0.001
	Post-adaptation	*W* = 0.736, *p* = 0.002	*W* = 0.622, *p* < 0.001	*W* = 0.605, *p* < 0.001	*W* = 0.758, *p* = 0.004	*W* = 0.413, *p* < 0.001	*W* = 0.606, *p* < 0.001

The normality results for the decay constant distribution in the sequential combination trials for the adaptation period correspond only to adaptation-2 of those experiments.

Friedman's test revealed that the difference in step time asymmetry was statistically significant (*p* < 0.05) between baseline, late adaptation, and early post-adaptation stages for all trials except $T_{Sc}$, as shown in Table 10. Wilcoxon signed-rank tests showed that changes in step time asymmetry during late adaptation and early post-adaptation were significant for $T_{C}$ and both congruent trials: $T_{SC}$ and $T_{CS}$. Results also showed that step time asymmetry was significantly different (*p* < 0.05) at late adaptation for $T_{S}$ and $T_{cS}$, but those effects were not significant during early post-adaptation (Table 11).

**Table 10:** Friedman's test results showing statistical differences in step time asymmetry among baseline, late adaptation, and early post-adaptation for the six trial types.

	$T_{S}$ *	$T_{C}$ *	$T_{SC}$ *	$T_{CS}$ *	$T_{Sc}$	$T_{cS}$ *
$\chi^{2}(2)$	15.8	14.6	9.6	16.8	5.0	11.4
*p*-value	<0.001	<0.001	0.008	<0.001	0.082	0.003

* Statistical significance at the 0.05 level is shown with an asterisk.

**Table 11:** Wilcoxon signed-rank results for step time asymmetry between (i) baseline and late adaptation, and (ii) baseline and early post-adaptation.

	$T_{S}$*	$T_{C}$**	$T_{SC}$**	$T_{CS}$**	$T_{cS}$*
Baseline vs. Late adaptation	*Z* = −2.803, *p* = 0.005	*Z* = −2.803, *p* = 0.005	*Z* = 2.395, *p* = 0.017	*Z* = −2.803, *p* = 0.005	*Z* = −2.701, *p* = 0.007
Baseline vs. Early post-adaptation	*Z* = −0.764, *p* = 0.445	*Z* = −2.701, *p* = 0.007	*Z* = −2.803, *p* = 0.005	*Z* = −2.395, *p* = 0.017	*Z* = −1.07, *p* = 0.285

Statistical significance at the 0.05 level for (i) is shown with an asterisk (*), and for both (i) and (ii) is demonstrated using two asterisks (**).

#### 3.4.1 Facilitation of adaptation to SBT and ARAC for step time asymmetry

Friedman's test showed that the difference in the implicit process among $T_{S}$, $T_{CS}$, and $T_{cS}$ was statistically significant: $\chi^{2}(2)\;=\;7.8,\;p\;=\;0.0202$ (distributions shown in Figure 9). Multiple pairwise comparisons were performed, as shown in Table 12. Friedman's test also revealed that the difference in the larger decay constant among $T_{S}$, $T_{CS}$, and $T_{cS}$ was not statistically significant: $\chi^{2}(2)\;=\;1.4,\;p\;=\;0.4966$.

**Table 12:** Wilcoxon signed-rank test results for the implicit adaptation rate of step time asymmetry.

	$T_{C}-T_{S}$	$T_{CS}-T_{S}$	$T_{cS}-T_{S}$*	$T_{cS}-T_{CS}$*
*Z*	−1.682	−0.153	−1.988	−1.988
*p*-value	0.093	0.878	0.047	0.047

The asterisk (*) indicates statistical significance (*p* < 0.05) in the difference between the decay constants associated with the second half of the adaptation process for the specified trial types.

The difference in the explicit adaptation component to ARAC among $T_{C}$, $T_{SC}$, and $T_{Sc}$ was statistically significant: $\chi^{2}(2)\;=\;6.2,\;p\;=\;0.045$ (distributions shown in Figure 9). The results of multiple pairwise comparisons between the corresponding decay constants of $T_{C}$, $T_{SC}$, and $T_{Sc}$ are outlined in Table 13. The difference in the decay constant indicative of the implicit adaptation rate (to ARAC) between $T_{C}$, $T_{SC}$, and $T_{Sc}$, was not statistically significant: $\chi^{2}(2)\;=\;5.4,\;p\;=\;0.0672$ (Figure 9).

**Table 13:** Results of multiple pairwise comparisons for the explicit adaptation rate to ARAC of step time asymmetry.

	$T_{C}-T_{S}$	$T_{SC}-\;T_{C}$*	$T_{Sc}-T_{C}$	$T_{Sc}-T_{SC}$
*Z*	−0.561	−1.988	−0.968	−1.682
*p*-value	0.575	0.047	0.333	0.093

The asterisk (*) indicates statistical significance (*p* < 0.05) in the difference between the explicit decay constants associated with the second half of the adaptation process for the specified trial types.

#### 3.4.2 Rate of de-adaptation for step time asymmetry

**Figure 10:**
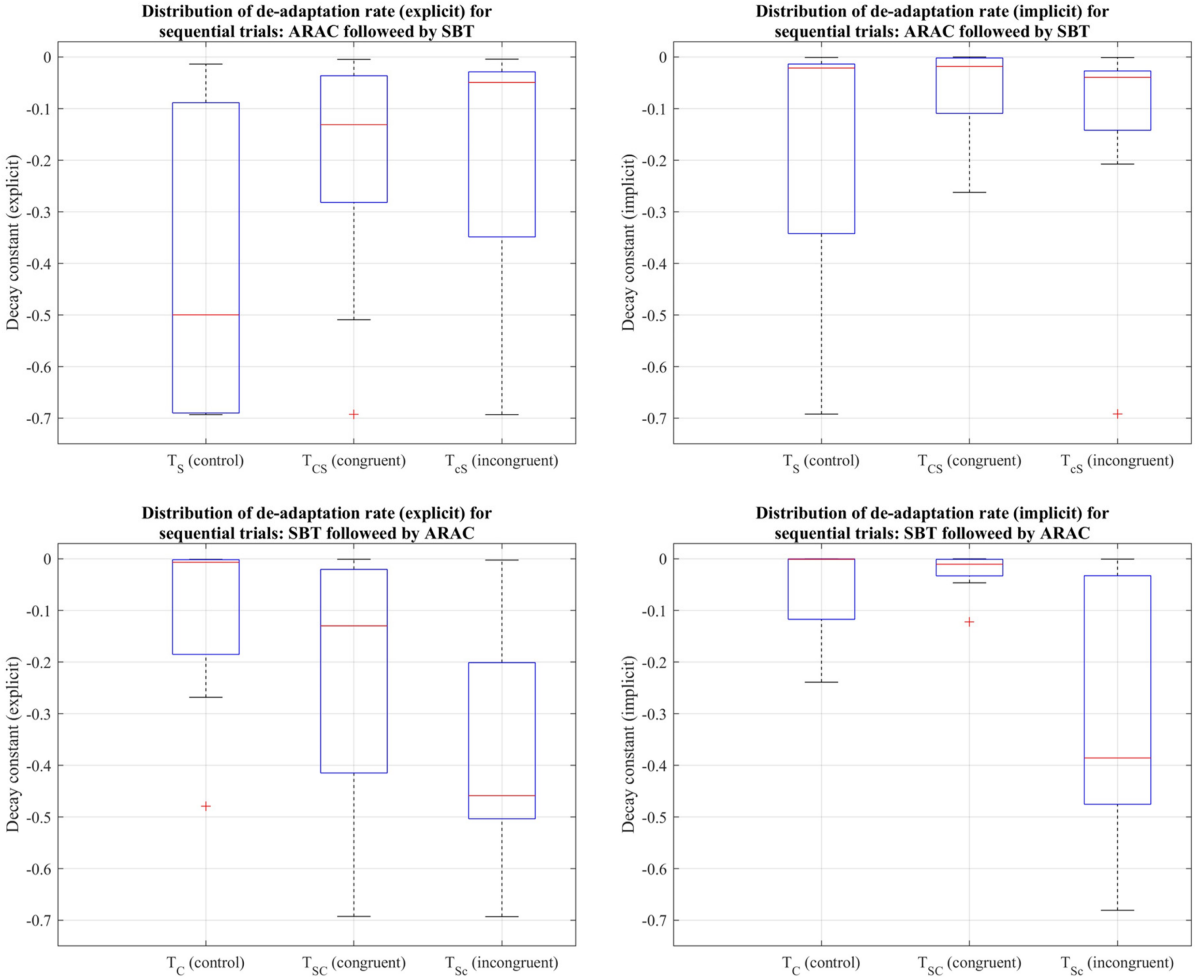
Distribution of decay constants depicting the explicit (left column) and implicit (right column) components of de-adaptation between the six trial types for step time asymmetry demonstrated using a box-and-whisker plot. The top row demonstrates distribution of de-adaptation rate from SBT and trials in which ARAC is followed by SBT; the bottom row demonstrates distribution of de-adaptation rate from ARAC and trials in which SBT is followed by ARAC. The box outlined in blue shows the interquartile range, the red horizontal line indicates the median of the distribution, and the outliers are shown using the red “+” symbols.

Friedman's test revealed that the difference in decay constants among the six trial types was not statistically significant for the explicit process: $\chi^{2}(5)\;=\;5.43,\;p\;=\;0.3658$, or the implicit process: $\chi^{2}(5)\;=\;7.37,\;p\;=\;0.1944$ (distributions shown in Figure 10).

## 4 Discussion

It is essential for gait rehabilitation techniques to account for sensory or cognitive deficits to facilitate personalized gait training in post-stroke individuals. For instance, if a post-stroke subject's cognitive ability is reduced, their gait rehabilitation is likely to benefit more from a technique such as SBT that relies less on their cognition for improved motor outcome. On the other hand, if a subject suffers from reduced proprioception, their physical therapist may consider a similar asymmetric gait intervention (e.g., ARAC) despite the higher cognitive burden it places on the individual. This study hypothesized that the temporary locomotor savings obtained from one context would facilitate the rate at which a healthy individual adapts to another context with similar locomotor demands. This may be attributed to the individual's ability to store information about their motor performance, also known as working motor memory (29). In other words, we hypothesized that working motor memory serves as a tool to enhance the rate of adaptation to a different gait intervention technique (30).

The de-adaptation processes from split-belt treadmill training and rhythmic cueing occur in distinct ways. Split-belt treadmill training is usually followed by a short period of readjustment once the perturbation is removed and subjects walk on a tied-belt treadmill. During this period, their gait asymmetry is briefly exaggerated on the contralateral limb. Results of this study showed that spatiotemporal asymmetries were significantly exaggerated during late adaptation and early post-adaptation stages (compared to baseline conditions) for $T_{C}$, $T_{S}$, and sequential combination trials ending with split-belt treadmill, i.e., $T_{CS}$ and $T_{cS}$.

Previous research has shown that altered cognitive load (i.e., exaggeration or inhibition of awareness) during split-belt treadmill training resulted in enhanced generalization of temporal gait features (31). Cognitive factors, such as attention, play a key role in facilitating the generalization of a learned motor behavior from one context to another (32). Post-stroke conditions are often associated with reduced cognitive and/or sensorimotor abilities (33–35), which results in a reduction in the individual's adaptability to certain gait intervention techniques. In addition to improved generalizability for split-belt treadmill training, sequentially applying gait interventions was also theorized to accelerate the motor adaptation process in a way that would account for their post-stroke motor learning deficits.

### 4.1 Computational models for locomotor learning in gait rehabilitation

The superiority of a double-component exponential model over a single-component model (12, 13) was established for locomotor adaptation and de-adaptation to two forms of asymmetric gait intervention techniques (8, 24) for asymmetries in step length and step time. Each adaptation model generated two decay constants indicative of the rate of adaptation or the rate at which acquired gait asymmetry was lost, i.e., de-adaptation rate(s). This finding aligns with a prior study suggesting that the dual adaptation process likely relies on the same error to guide both explicit and implicit processes (36). In our study, explicit adaptation (fast) was driven by either proprioceptive or predictive error in the form of feedback (sensorimotor adaptation) or feedforward mechanisms (instructional adaptation) (37, 38), and implicit adaptation (slow) process was primarily driven by use-dependent repetition. It must also be noted that the implicit process was also likely to be driven by multiple adaptation paradigms, with distinct psychological drivers and neural substrates that correspond to them (3, 14). In future studies, the applicability of other computational models such as the power-law model may also be compared against the double-component exponential model for increased confidence in the estimation of relevant time constants (9).

### 4.2 Spatiotemporal gait characteristics

Sequentially applying two interventions has the potential to accelerate the adaptation process (to the second intervention) while reducing the rate of de-adaptation. Other gait rehabilitation protocols that predominantly engage sensorimotor adaptation (e.g., robot-assisted therapy) or instructional adaptation processes (e.g., visual or tactile cueing) may incorporate these findings to facilitate adaptation or inhibit de-adaptation. Comparisons in decay constants between the control interventions $\left(T_{S},T_{C}\right)$ and their corresponding incongruent trials $\left(T_{cS},\;T_{Sc}\right)$ are discussed in Section 4.2.1, and comparisons of control interventions against their corresponding congruent trials $\left(T_{CS},\;T_{SC}\right)$ are discussed in Section 4.2.2.

#### 4.2.1 Adaptation to intervention with misaligned (incongruent) temporal motor demands

##### 4.2.1.1 Step length

For step length, the difference among the control trials $\left(T_{S}\right)$ and their corresponding incongruent sequential combinations $\left(T_{cS}\right)$ was statistically significant only for the decay constant signifying the explicit rate of adaptation to split-belt treadmill. However, the explicit re-adaptation rate to asymmetric rhythmic auditory cueing did not exhibit any significant changes when following split-belt treadmill incongruently. This is consistent with a previous study that had found significant effects on the re-adaptation rate to split-belt treadmill using distraction or awareness techniques, demonstrating the effects of altered attention on its generalizability (31). This shows that the effects of different explicit adaptation mechanisms are not equivalent when engaged sequentially.

In addition, the difference in the implicit adaptation rate was statistically insignificant among the control trials and their sequential combinations for split-belt treadmill as well as asymmetric rhythmic auditory cueing. This lack of significant difference in the implicit adaptation process was somewhat anticipated, given that the implicit process is speculated to be involve multiple simultaneous processes with different timescales and duration of efficacy (14). In future studies, additional exponential components may be incorporated to assess the optimal number of exponents (representing different adaptation timescales) in modeling gait asymmetry.

##### 4.2.1.2 Step time

For step time, there were no statistically significant differences in the explicit rate of adaptation to split-belt treadmill among $T_{S}$, $T_{CS}$, and $T_{cS}$. This shows that instructional interventions (e.g., asymmetric rhythmic auditory cueing) have little to no effect on the explicit adaptation rate pertaining to sensorimotor/proprioceptive interventions (e.g., split-belt treadmill) in the temporal domain. The effects of interference appear to be not only task-specific but also specific to the motor performance measure (i.e., step length and step time).

The implicit component of adaptation to asymmetric rhythmic auditory cueing was not substantially affected when it was applied after split-belt treadmill. In contrast, the median (implicit) rate of adaptation to split-belt treadmill training was significantly reduced in both $T_{CS}$ and $T_{cS}$ compared to $T_{S}$. The implicit re-adaptation rate of step time asymmetry was significantly smaller during split-belt treadmill training when it was following asymmetric rhythmic cueing incongruently, i.e., opposite lateral equivalence between interventions. This shows that the effect of anterograde interference in the implicit rate of adaptation to split-belt treadmill was quite significant when the desired motor output was not aligned between split-belt treadmill training and asymmetric rhythmic auditory cueing.

##### 4.2.1.3 Interference affects rate of re-adaptation

The effect of interference on the rate of re-adaptation was a significant factor. This implies that the effectiveness of working motor memory transfer from one intervention to another was not only context-dependent and task-specific, but it was also different for the explicit and implicit adaptive processes for temporal aspects of a healthy individual's gait pattern.

#### 4.2.2 Congruence: temporal motor alignment between interventions

##### 4.2.2.1 Step length

For step length asymmetry, the rate of explicit adaptation to the split-belt treadmill was increased in the corresponding congruent combination (i.e., perfect motor alignment in interlimb asymmetry between the two interventions) when it was applied immediately after asymmetric rhythmic auditory cueing. This was demonstrated by the difference in explicit decay constant between $T_{S}$ and $T_{CS}$. Nine out of ten subjects exhibited an increased rate of adaptation to split-belt treadmill when it was applied subsequently after asymmetric rhythmic auditory cueing. Working motor memory obtained from adaptation to asymmetric rhythmic cues (associated with a higher cognitive load) was transferred to a different context/intervention that had a lower cognitive demand (i.e., split-belt treadmill) when their desired locomotor patterns were identical. In contrast, eight out of ten subjects showed a marked reduction in the rate of adaptation (explicit component) to asymmetric rhythmic auditory cueing when it was following split-belt treadmill training in the congruent condition.

##### 4.2.2.2 Step time

Step time asymmetry demonstrated similar trends (as step length) in the explicit adaptation rate to asymmetric rhythmic auditory cueing when it was applied subsequently after split-belt treadmill training. Inhibition of the explicit motor adaptation process may be attributed to the effects of anterograde interference on working motor memory.

##### 4.2.2.3 Anterograde interference

Anterograde interference is the inhibition of learning a new task when it is being executed soon after a different task (39). This learning phenomenon generally applies to the execution of two different tasks, but its relevance within different *adaptation mechanisms* (rather than different tasks) has not been explored. Previous studies that investigated reaching tasks found similar outcomes where the adaptation rate was reduced due to different timescales in the process of motor adaptation (40). The lack of statistical significance in the difference between the decay constants of $T_{Sc}$ and $T_{C}$ shows that there was no significant effect on the rate of adaptation to asymmetric rhythmic auditory cueing when it was applied incongruently after split-belt treadmill, suggesting that the interference factor was only significant when both interventions (and corresponding adaptation mechanisms) had the same desired motor output. In other words, the effects of anterograde interference on explicit adaptation are likely to be task-specific. Anterograde interference was only observed when the walking context moved from split-belt treadmill to asymmetric rhythmic auditory cueing: walking to rhythmic cues may help facilitate one's explicit adaptation to split-belt treadmill, but asymmetric walking on a split-belt treadmill would inhibit the rate of explicit adaptation to rhythmic cues. The results of this type of interference have significant effects on the adaptation rate, necessitating combination therapies to account for maximizing performance outcomes as well as the resulting adaptation/de-adaptation rate(s).

In summary, explicit adaptation to asymmetric rhythmic auditory cueing was significantly smaller when applied after split-belt treadmill, but this did not hold when the order was reversed. This shows evidence of the explicit motor adaptation process being unequally affected by different adaptation mechanisms and the way they are combined. These outcomes demonstrate the interaction effects between (i) adaptation mechanisms: instructional and proprioceptive, and (ii) the nature of perturbation or task-specificity between motor interventions, i.e., congruent and incongruent combinations, as they apply to motor interference and savings of step time asymmetry. In addition, motor memory consolidation is likely to have distinct attributes between different therapies and should be accounted for in the investigation of combined gait therapies.

### 4.3 Limitations and future work

Sensorimotor adaptation and instructional adaptation were presumed to be the predominant motor adaptation processes for split-belt treadmill and asymmetric rhythmic auditory cueing, respectively (3). The limiting effects of this presumption are likely to affect the modeling technique(s), and, consequently, have repercussions on the statistical assessment. This was exacerbated by the implicit process likely being a culmination of several adaptation processes with different timescales (14). Additional psychophysiological and neurological evidence may be required to confirm this supposition.

A lack of compliance in the subjects’ adaptation to asymmetric rhythmic auditory cueing was anticipated and is an inherent limitation of most instructional interventions. Future studies may involve the assessment of the behavior of compliant and non-compliant individuals separately to determine whether there are any differences in the way motor behavior is translated from one context to another. The outcomes of this proposed study would also help establish a relationship between compliance to rhythmic cues and its association with the individual's adaptability to split-belt treadmill.

Additionally, the heterogeneity in gait response to rhythmic cues resulted in a broader distribution of their decay constants relative to the SBT training condition. This is likely to increase the randomness of the decay constants in the fitted models, thereby reducing statistical power in the set of comparisons. In addition, the optimization and modeling procedures are quite vulnerable to local minima, which can vary substantially among subjects. The particle swarm optimization technique is extremely sensitive to slight perturbations in gait asymmetry. This means that if an individual decided to stop complying with the rhythmic cues during their ongoing experiment, it would have significant effects on the accuracy of the outcome. In addition, a larger sample size would enhance the generalizability of the findings to a broader range of individuals and improve its reliability by reducing the impact of random variability. Future studies may explore additional factors that may influence the subjects’ level of response, e.g., individual rhythm abilities.

The applicability of different intervention techniques and their combinations during different stages of post-stroke recovery must also be investigated to establish targeted physical therapy. An increased adaptation rate is conducive to targeted gait intervention techniques, especially for post-stroke individuals with impaired cognitive and/or sensorimotor adaptation abilities. Using an intervention that can benefit them more immediately may help facilitate their adaptation to another intervention that targets their gait asymmetries more accurately. If a future study's outcomes reveal that the rate of adaptation is not improved for post-stroke individuals based on the outcomes of our study, testing similar sequentially combined interventions would help locate the limitations of the ability of post-stroke subjects to adapt using certain processes, and their ability (or lack thereof) to generalize those motor memories in other contexts. Different forms of asymmetric rhythmic cueing, such as tactile or visual, may also be utilized to study how different sensory modalities can affect the adaptation process.

## 5 Conclusions

The outcomes show clearly that the explicit adaptation rate to instructional interventions (e.g., rhythmic cueing) was inhibited when it was following a similar intervention that engaged proprioceptive adaptation techniques (e.g., split-belt treadmill) to impose the same motor output for step length and step time. Interestingly, the explicit rate of adaptation to split-belt treadmill was facilitated when it was following asymmetric rhythmic auditory cueing for step length. The reason behind this may be attributed to phenomena such as anterograde interference, and additional physiological evidence such as neural activation patterns would be needed to form definitive conclusions. To summarize, this sequence (ARAC → SBT) is likely to be beneficial to a stroke subject that shows an ability to respond to ARAC and also has retained their proprioceptive abilities.

Contrary to the trends observed for the explicit rate of adaptation, the implicit rate of adaptation to split-belt treadmill was impeded when following incongruent asymmetric rhythmic auditory cueing, which indicates that the two simultaneous adaptation processes (explicit and implicit) have distinct timescales and interact differently with instructional interventions. In addition, the rate of implicit adaptation to the split-belt treadmill was significantly greater for the congruent combination compared to the incongruent counterpart, suggesting facilitated re-adaptation (implicit) resulting from similar motor memory from the preceding intervention, i.e., asymmetric rhythmic cueing. These interactions are also dependent on congruence between the two interventions. Overall, the facilitation (or inhibition) of motor adaptation shows distinct characteristics between the explicit and implicit processes. In addition, the effects on the adaptation rate are context-specific (i.e., sequence) and task-specific (i.e., congruence). Future works may also investigate the characteristics of other adaptation mechanisms and the way they interact in motor memory consolidation.

## Data Availability

The original contributions presented in the study are included in the article. Further inquiries can be directed to the corresponding author.
