# Supplementary figures and images for: Facilitation of motor adaptation using multiple gait rehabilitation interventions
Source: Front Rehabil Sci. 2024 Oct 10;5:1238139. doi: 10.3389/fresc.2024.1238139 (PMC11499191; doi:10.3389/fresc.2024.1238139)

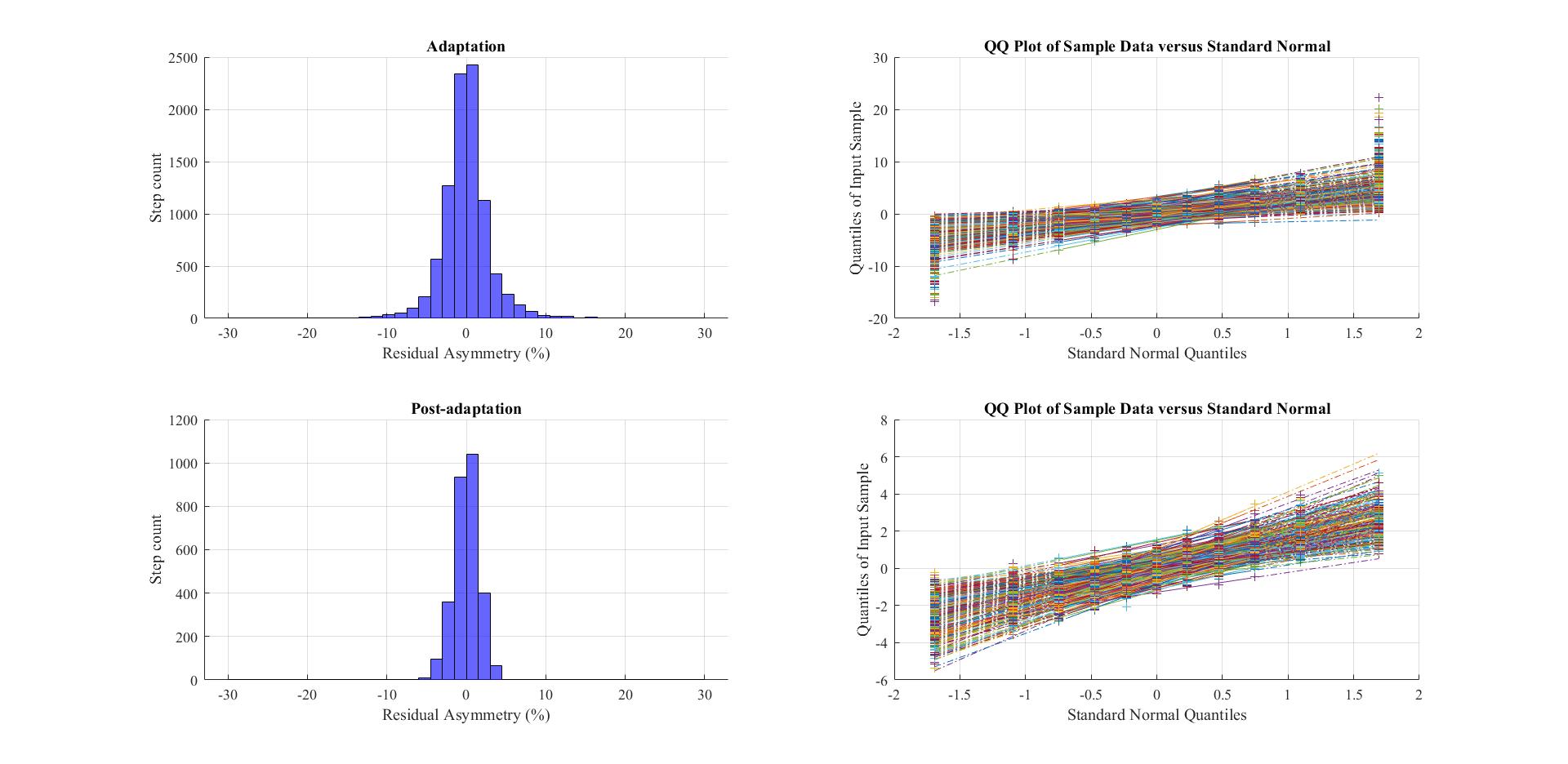

Supplement: Supplementary file 3 [file Datasheet1.zip › residual/SLA/SLA_T-C_double.jpg]

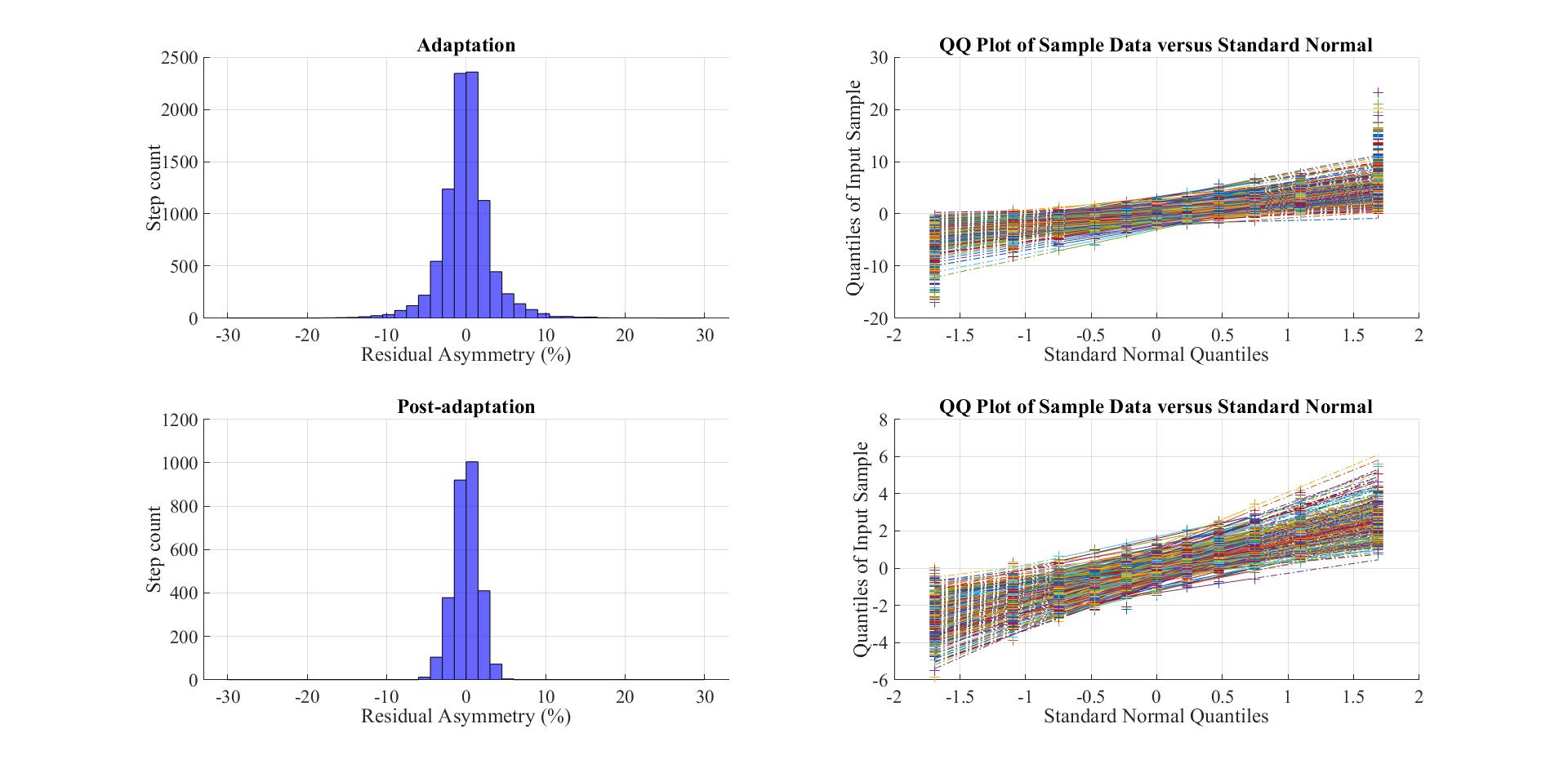

Supplement: Supplementary file 3 [file Datasheet1.zip › residual/SLA/SLA_T-C_single.jpg]

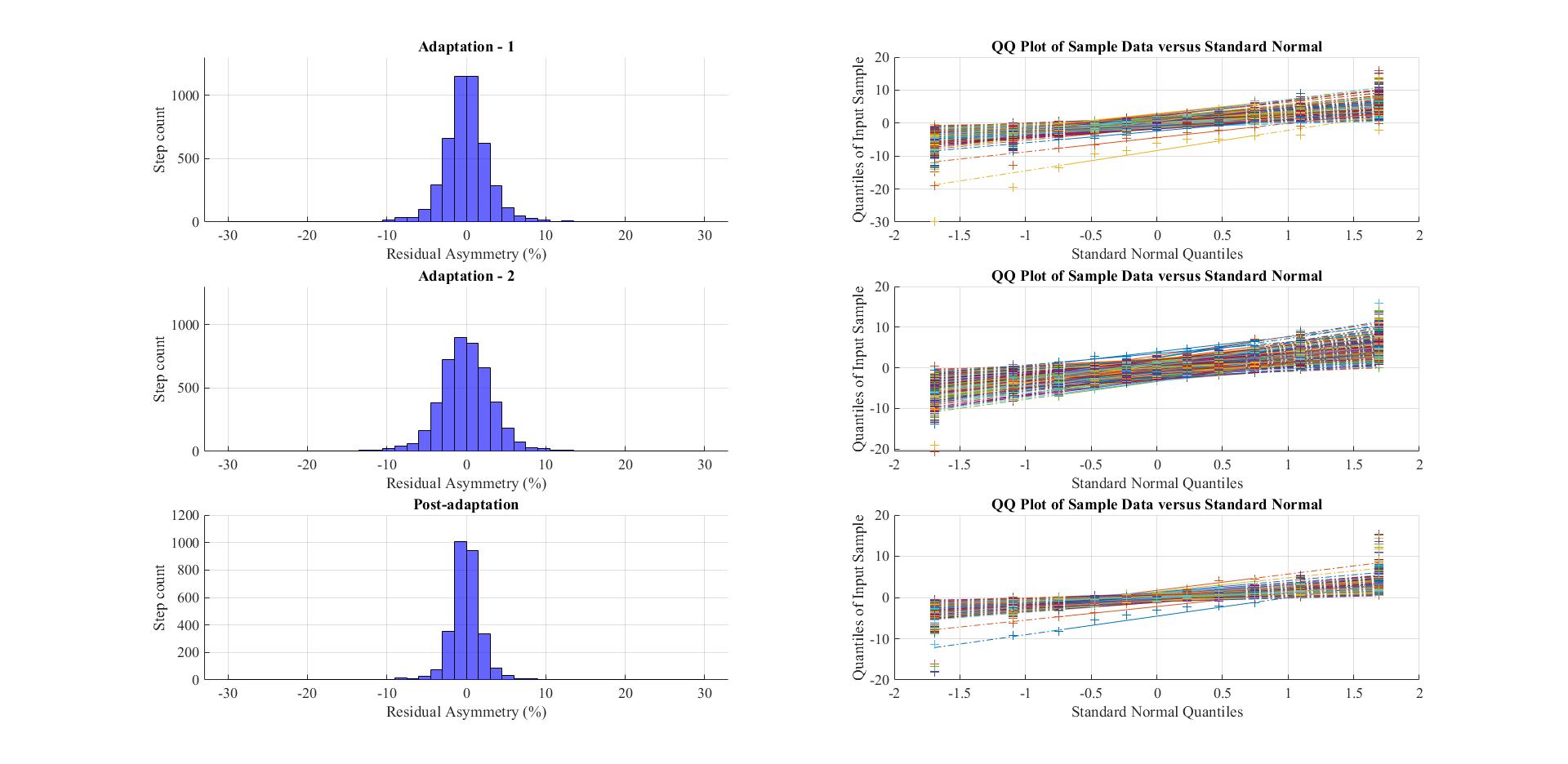

Supplement: Supplementary file 3 [file Datasheet1.zip › residual/SLA/SLA_T-CS_double.jpg]

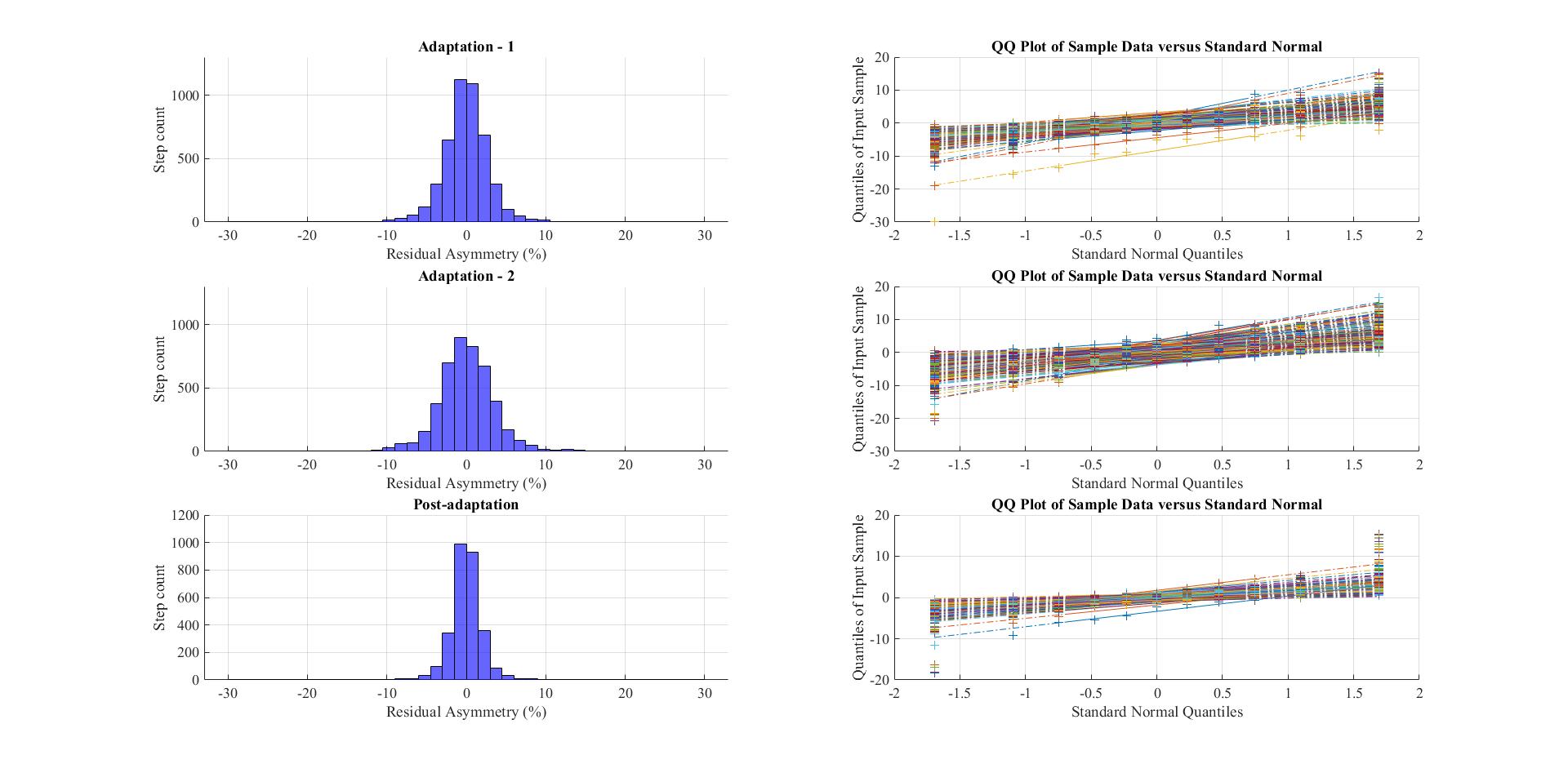

Supplement: Supplementary file 3 [file Datasheet1.zip › residual/SLA/SLA_T-CS_single.jpg]

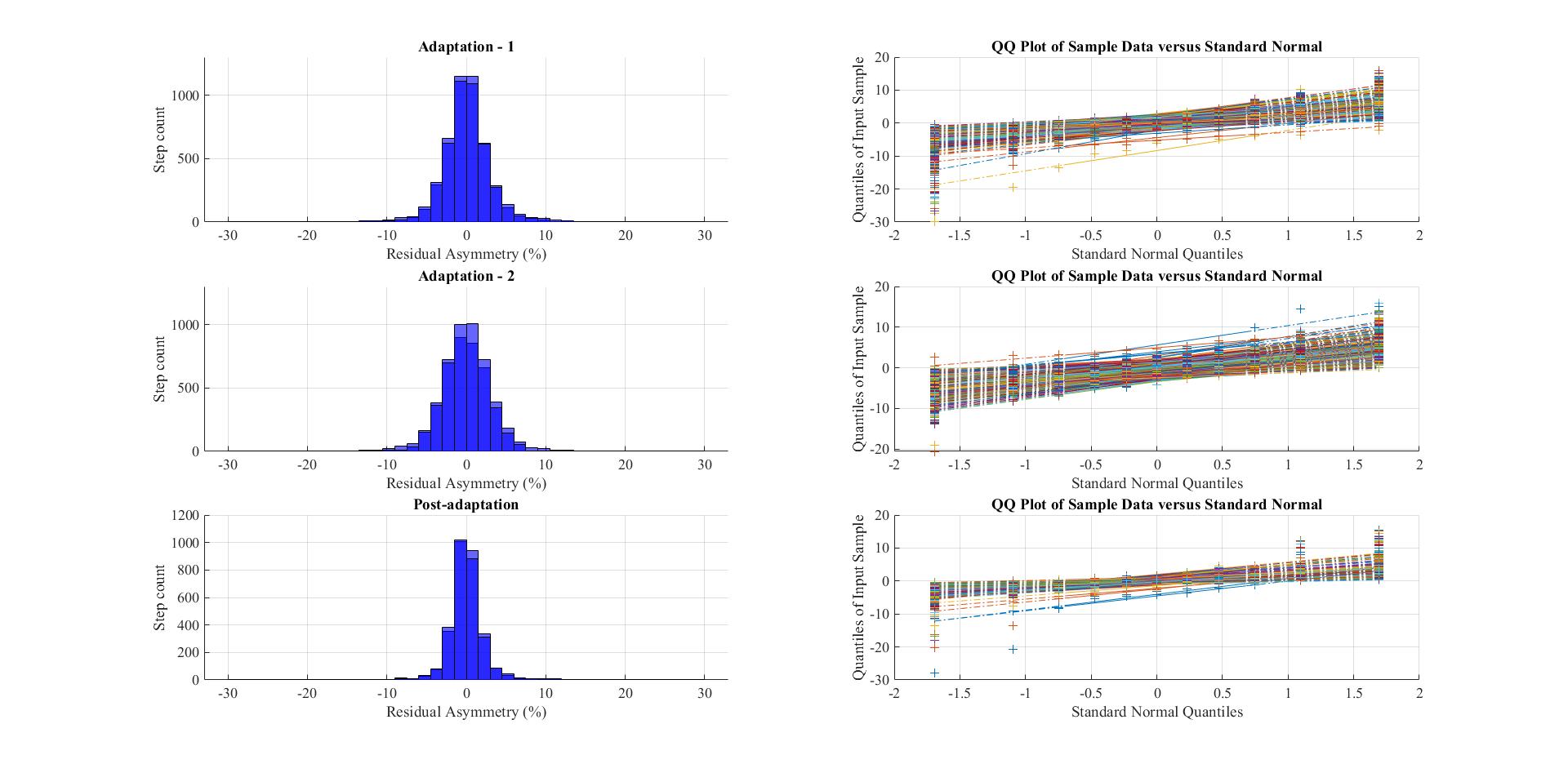

Supplement: Supplementary file 3 [file Datasheet1.zip › residual/SLA/SLA_T-cSI_double.jpg]

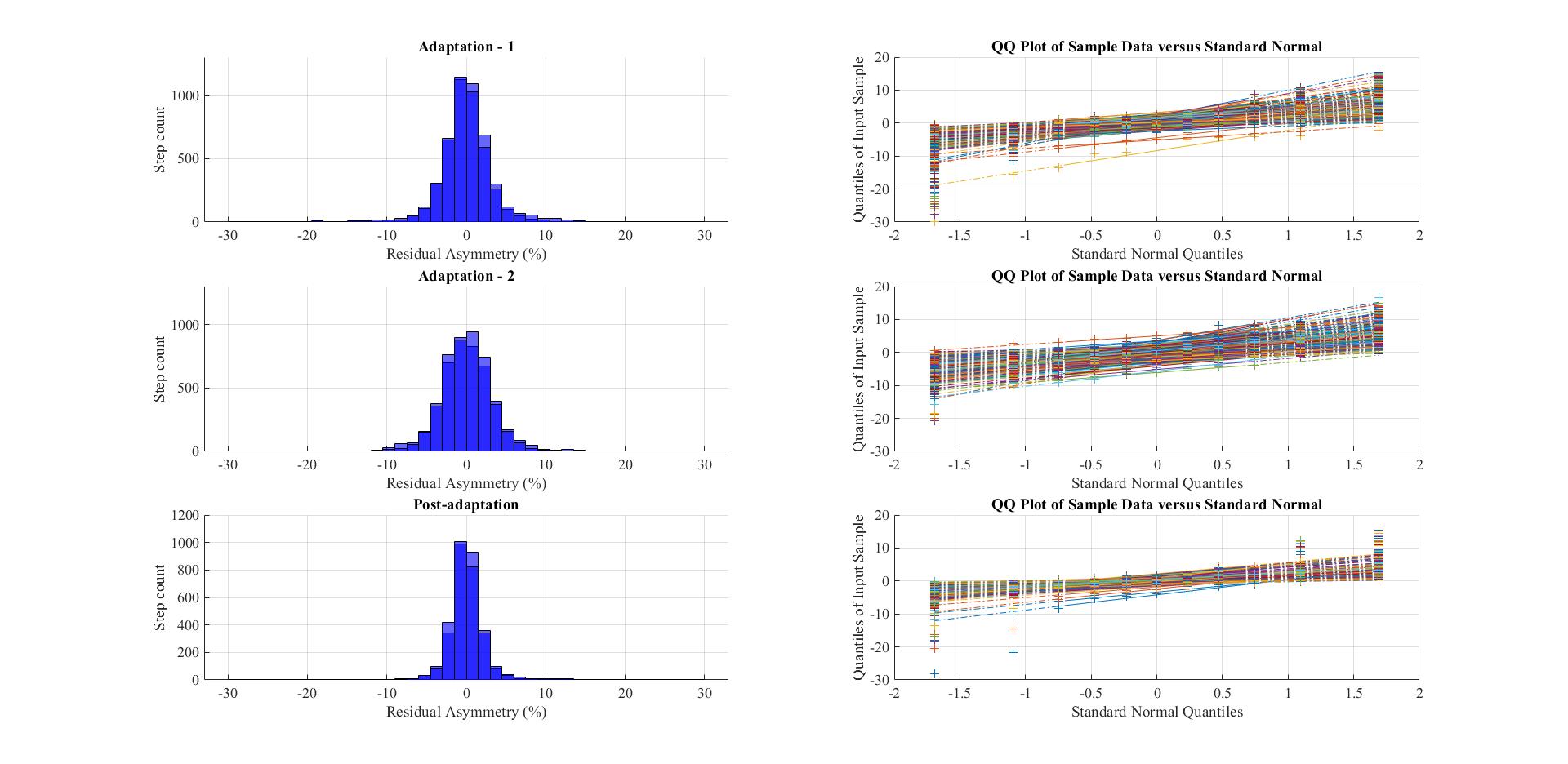

Supplement: Supplementary file 3 [file Datasheet1.zip › residual/SLA/SLA_T-cSI_single.jpg]

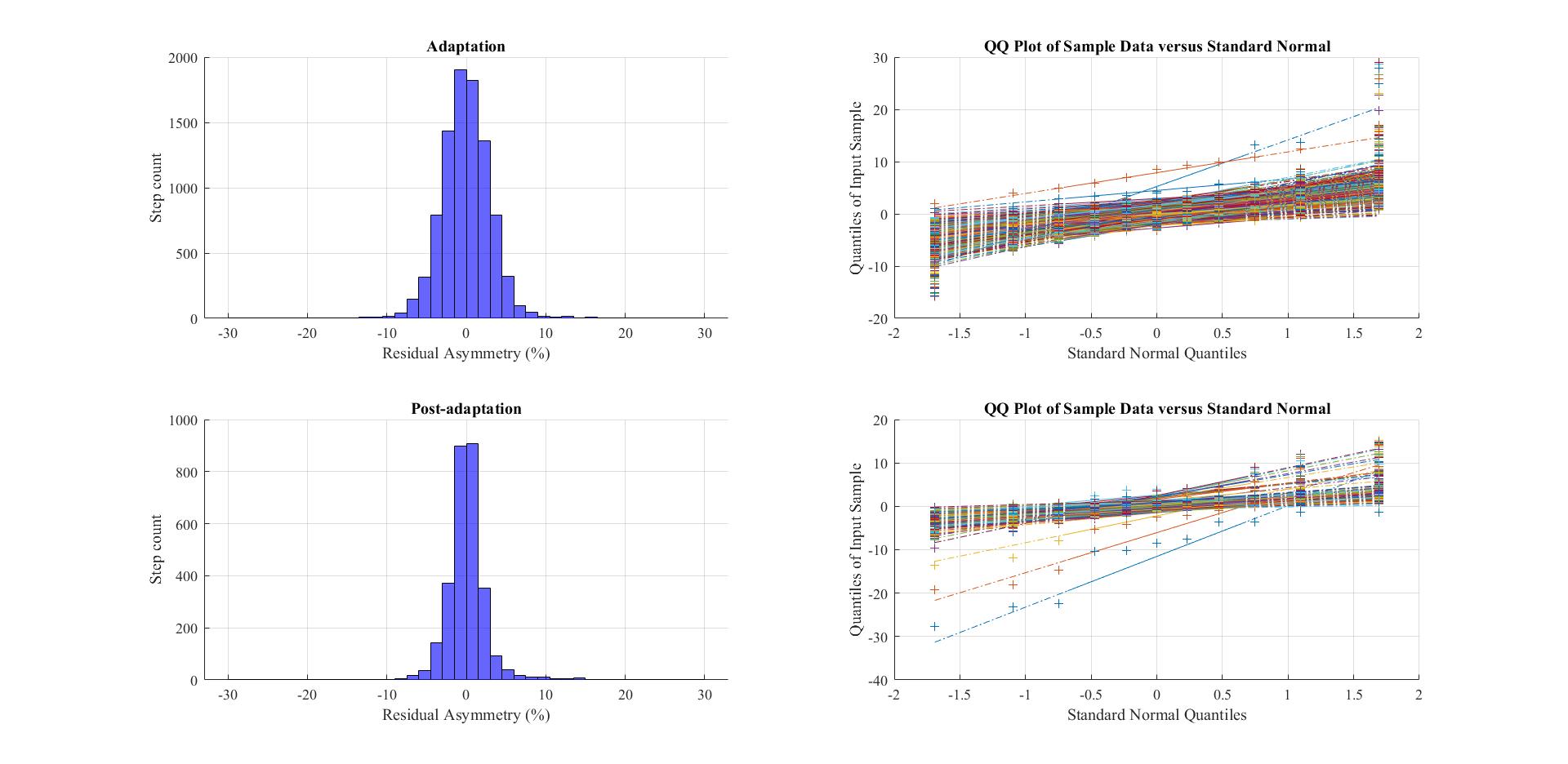

Supplement: Supplementary file 3 [file Datasheet1.zip › residual/SLA/SLA_T-S_double.jpg]

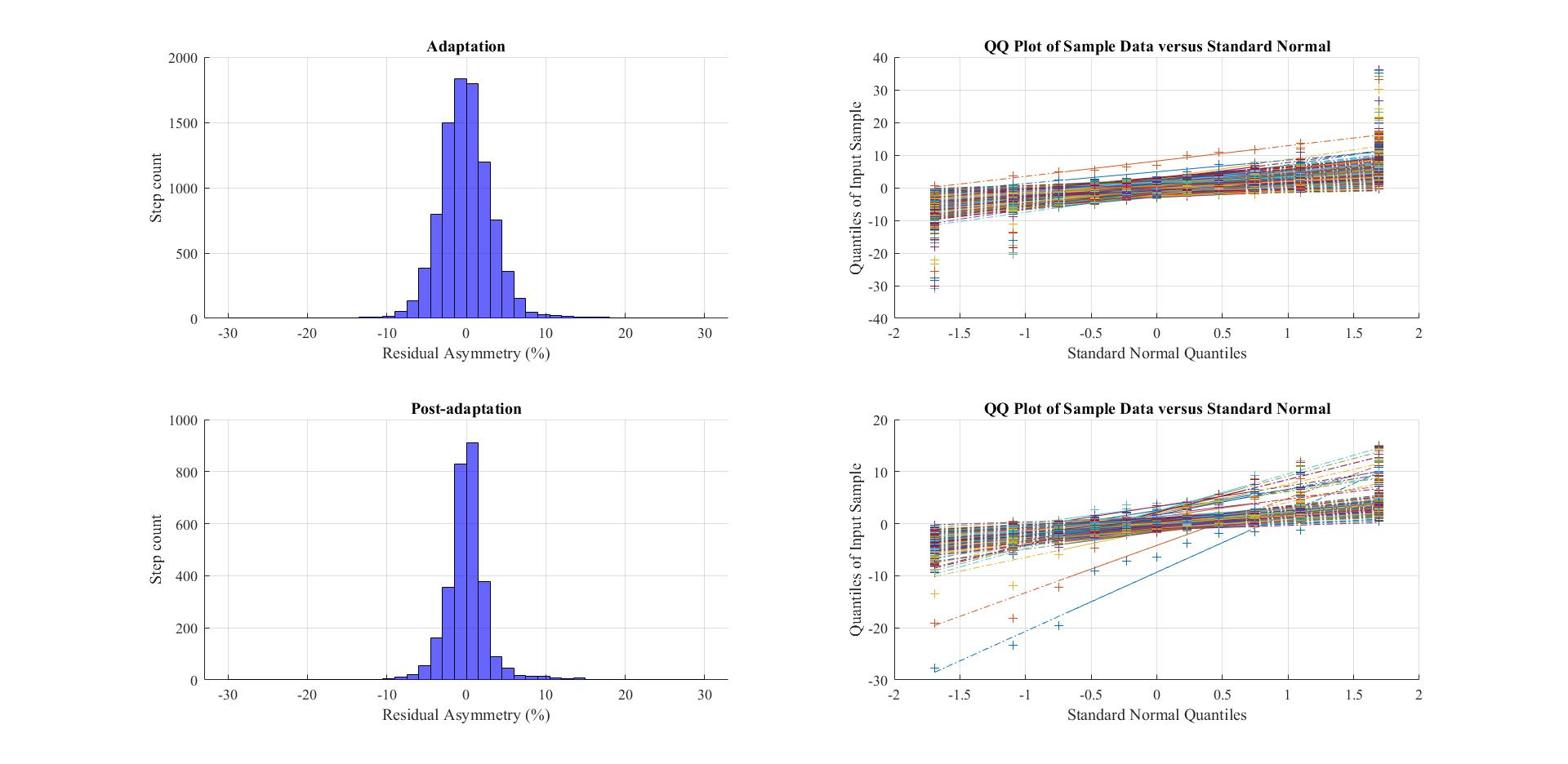

Supplement: Supplementary file 3 [file Datasheet1.zip › residual/SLA/SLA_T-S_single.jpg]

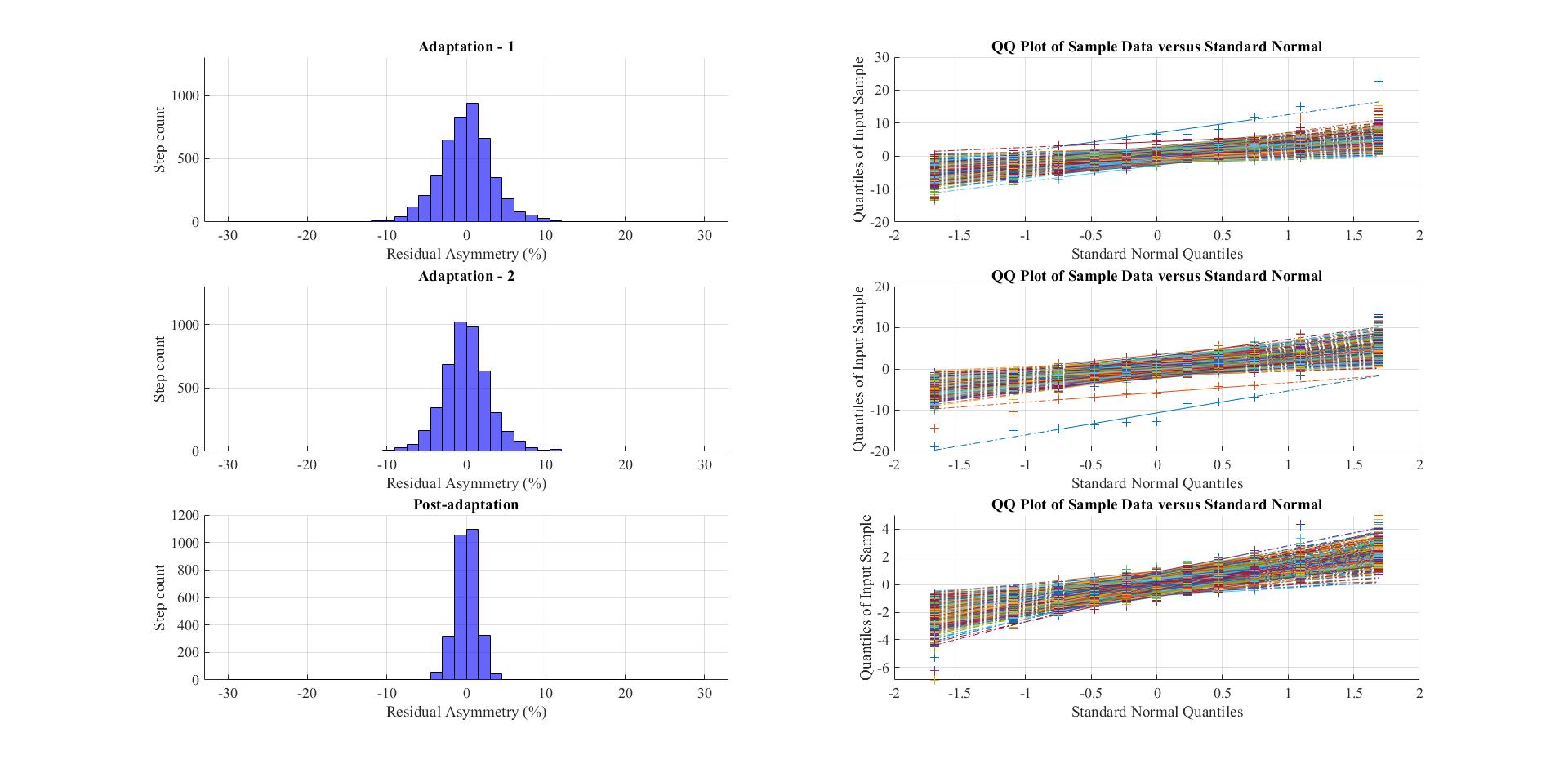

Supplement: Supplementary file 3 [file Datasheet1.zip › residual/SLA/SLA_T-SC_double.jpg]

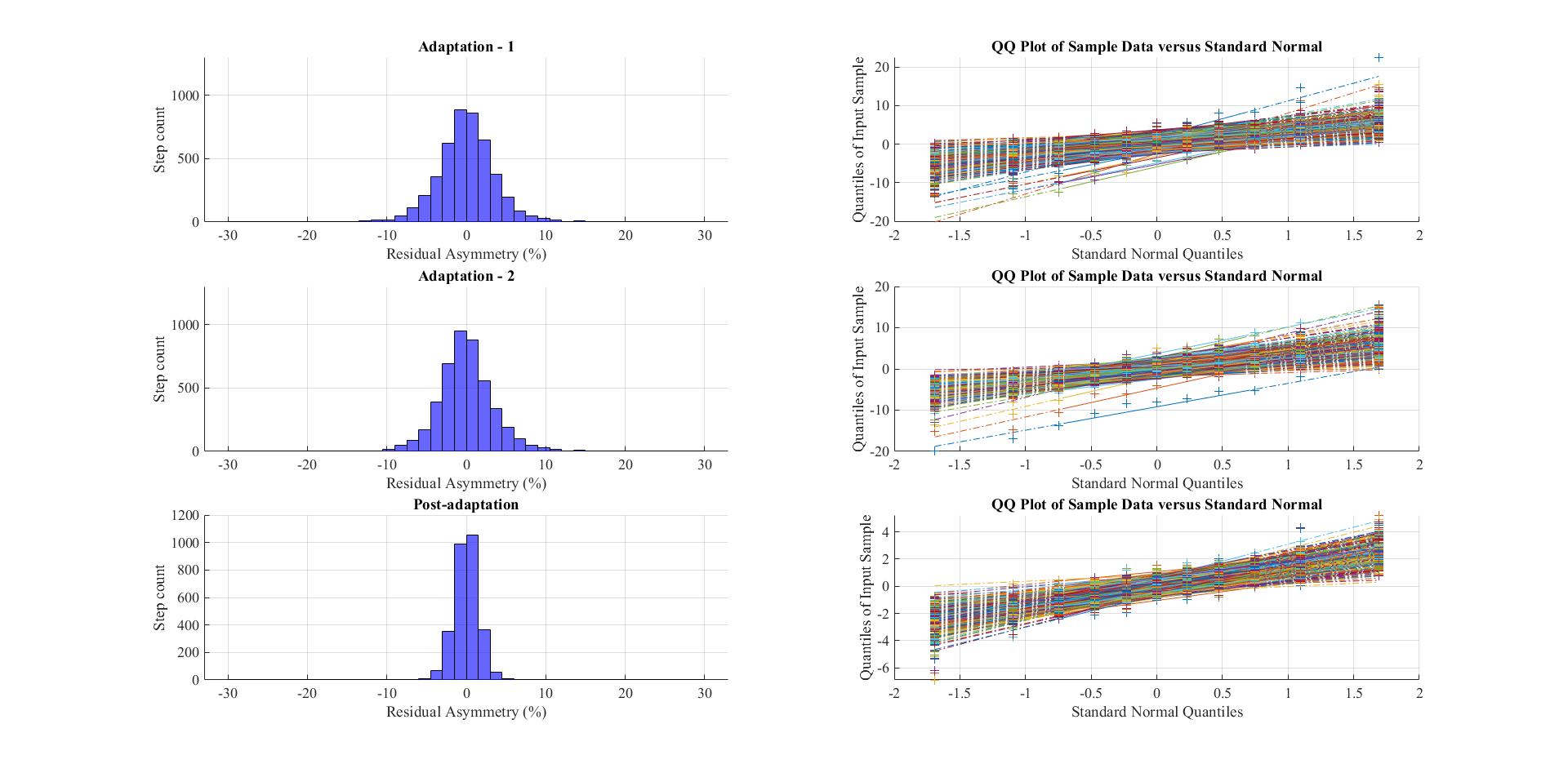

Supplement: Supplementary file 3 [file Datasheet1.zip › residual/SLA/SLA_T-SC_single.jpg]

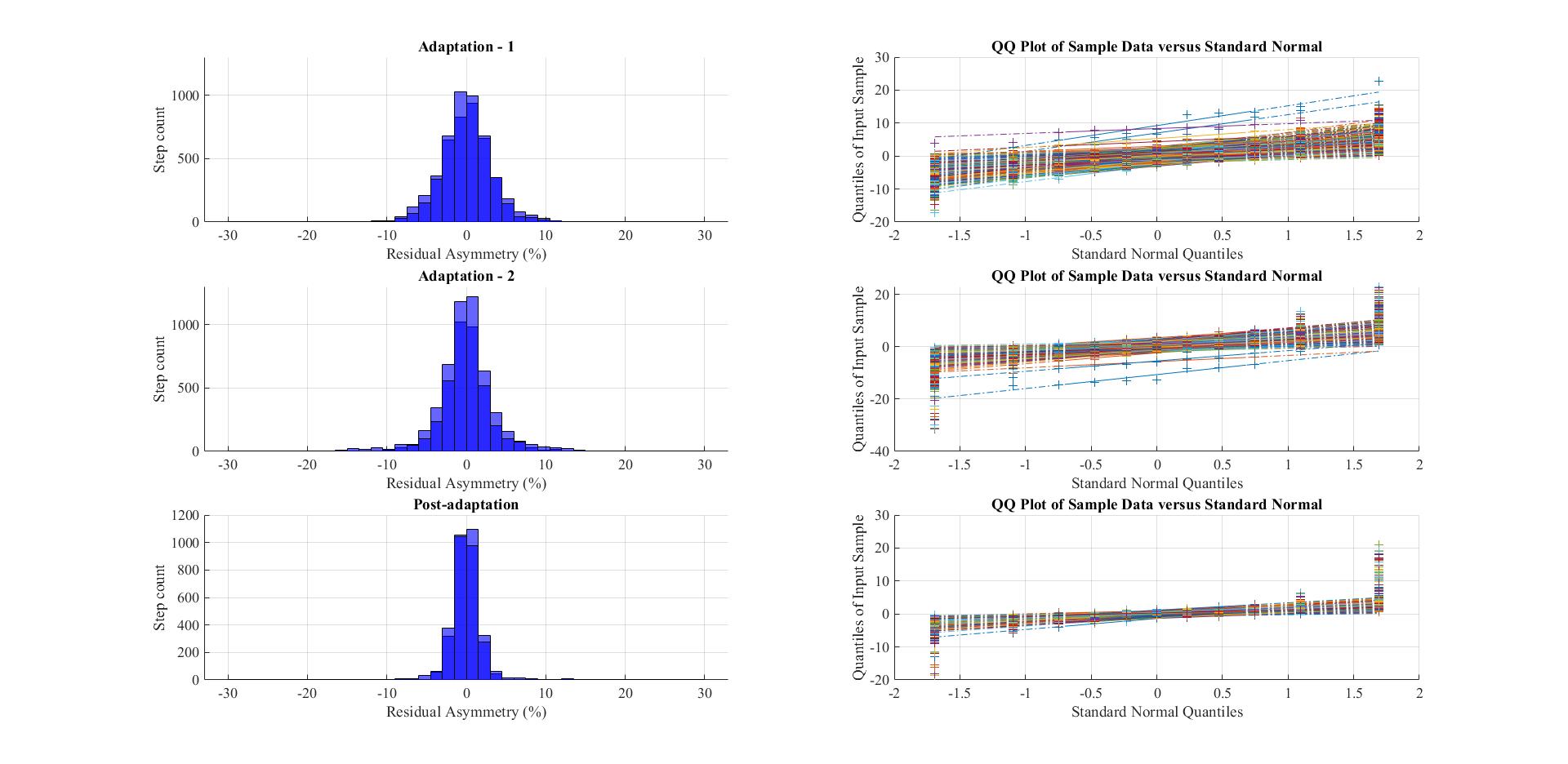

Supplement: Supplementary file 3 [file Datasheet1.zip › residual/SLA/SLA_T-ScI_double.jpg]

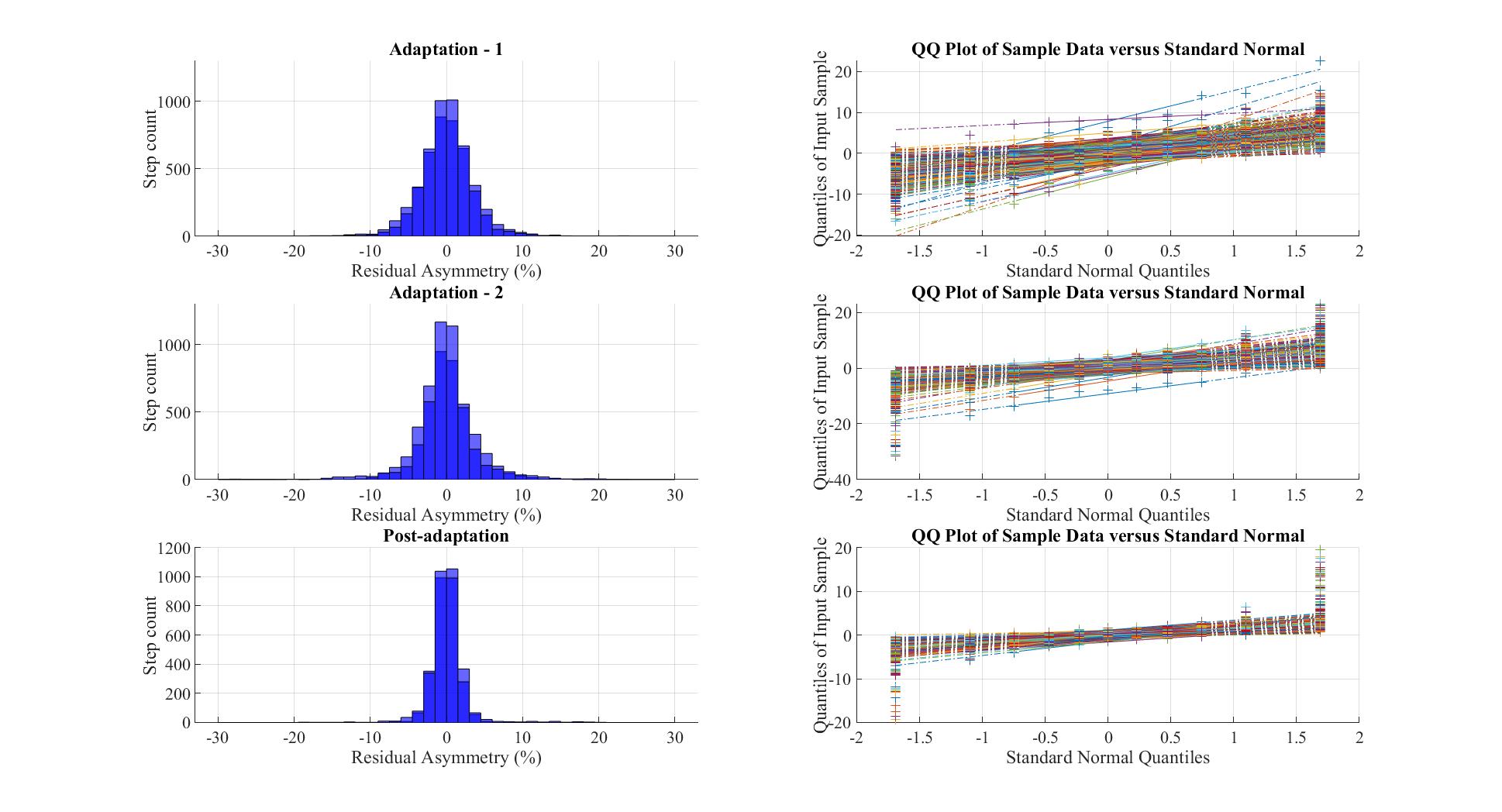

Supplement: Supplementary file 3 [file Datasheet1.zip › residual/SLA/SLA_T-ScI_single.jpg]

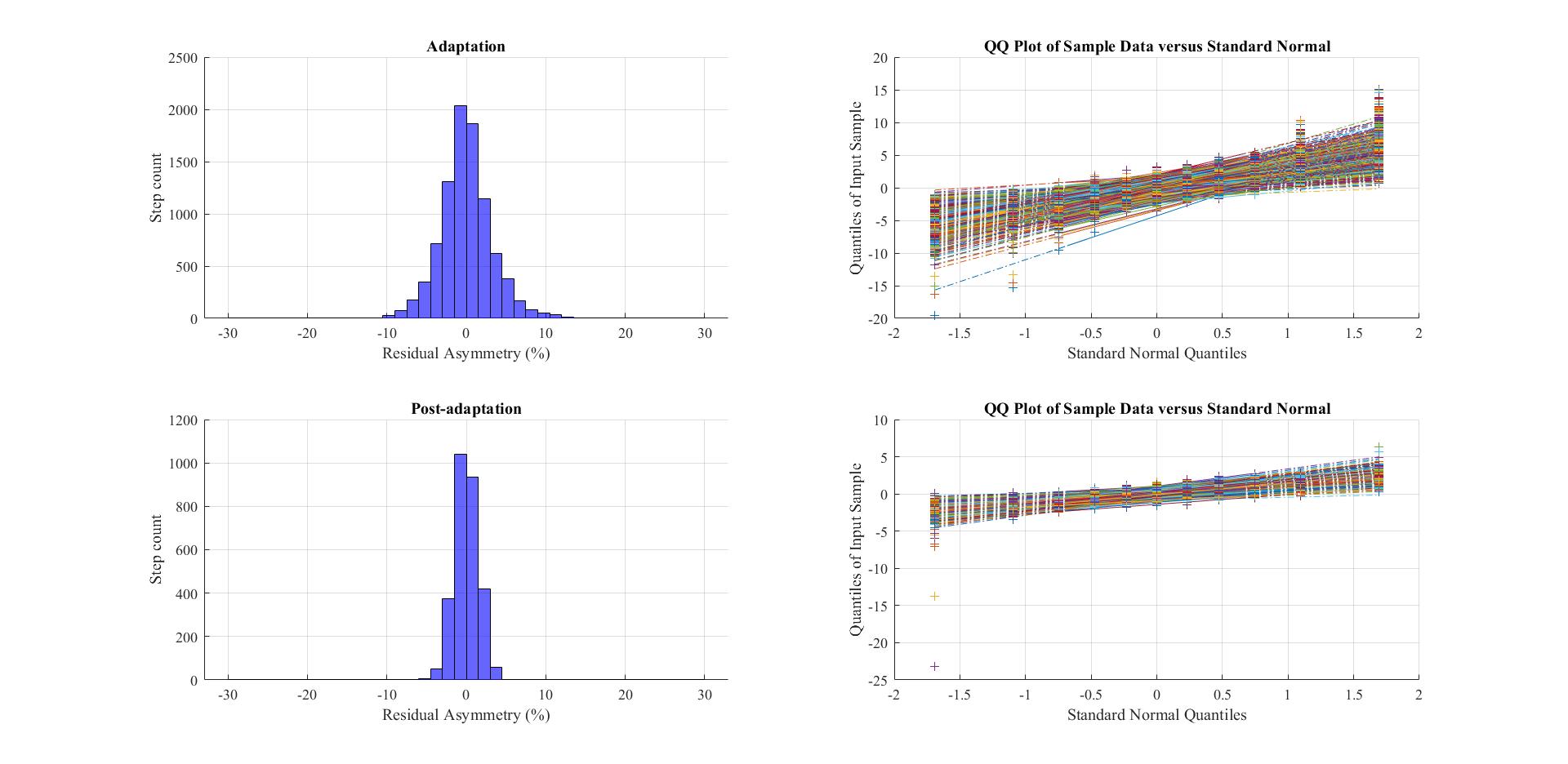

Supplement: Supplementary file 3 [file Datasheet1.zip › residual/STA/STA_T-C_double.jpg]

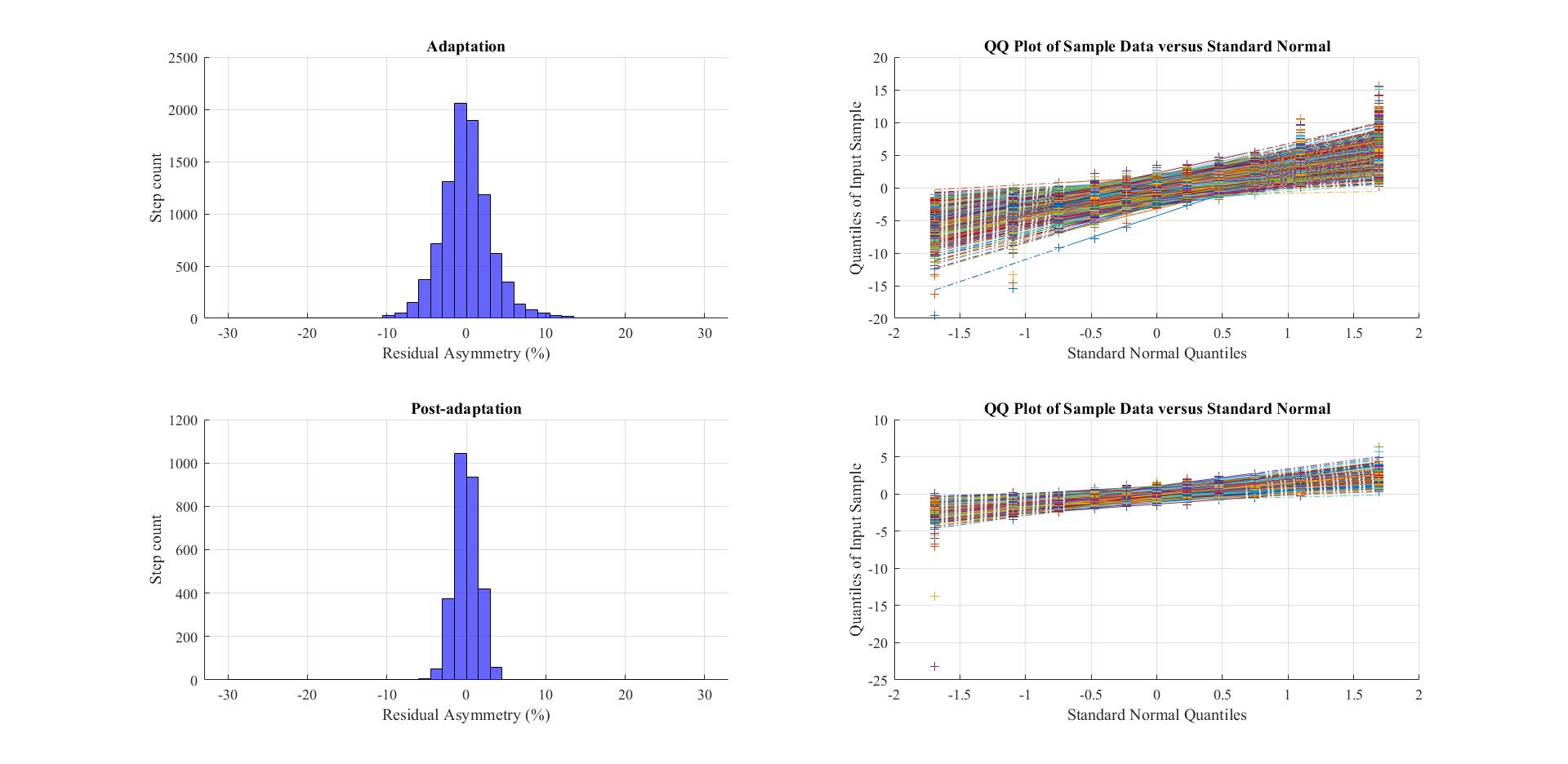

Supplement: Supplementary file 3 [file Datasheet1.zip › residual/STA/STA_T-C_single.jpg]

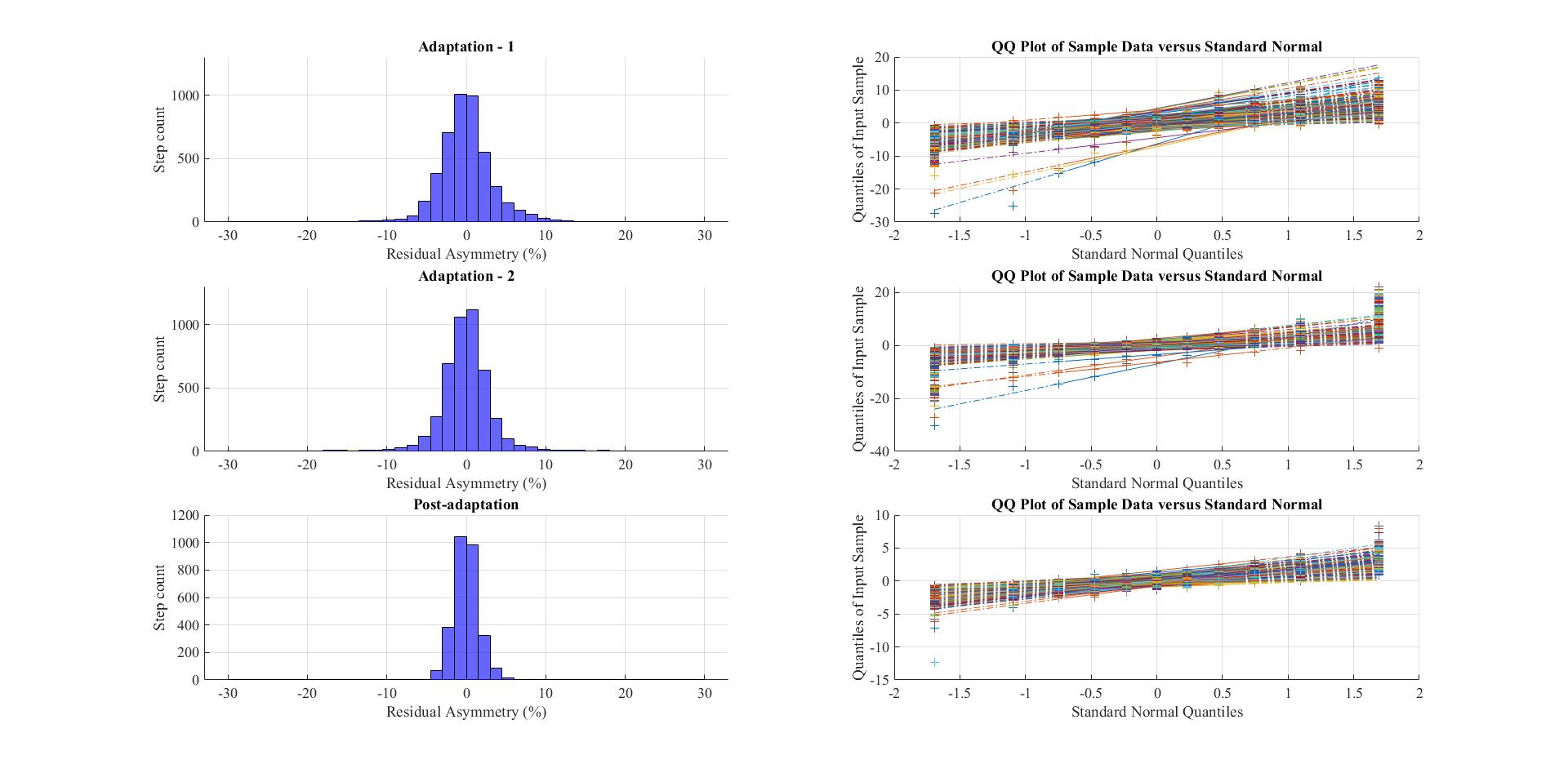

Supplement: Supplementary file 3 [file Datasheet1.zip › residual/STA/STA_T-CS_double.jpg]

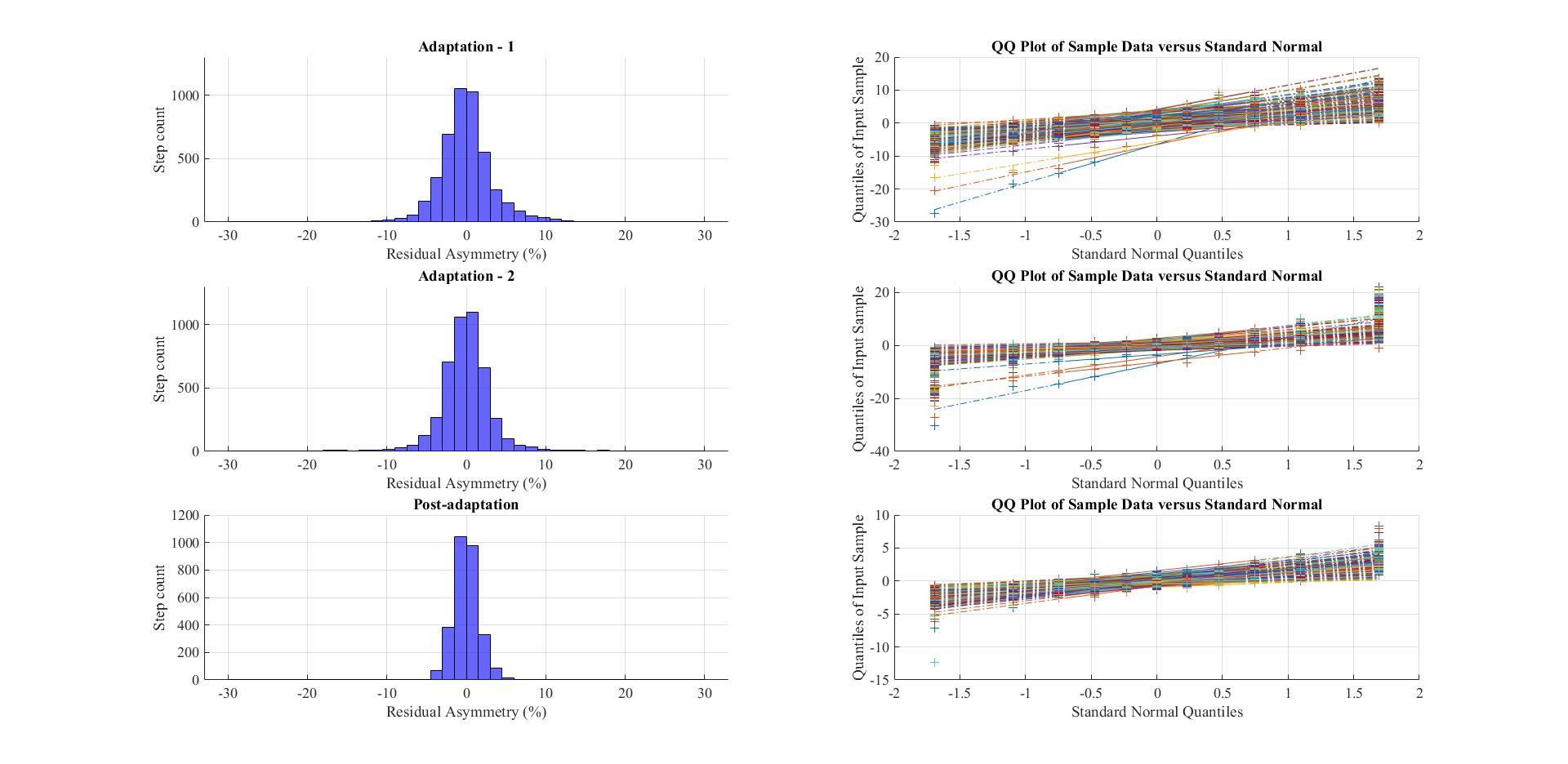

Supplement: Supplementary file 3 [file Datasheet1.zip › residual/STA/STA_T-CS_single.jpg]

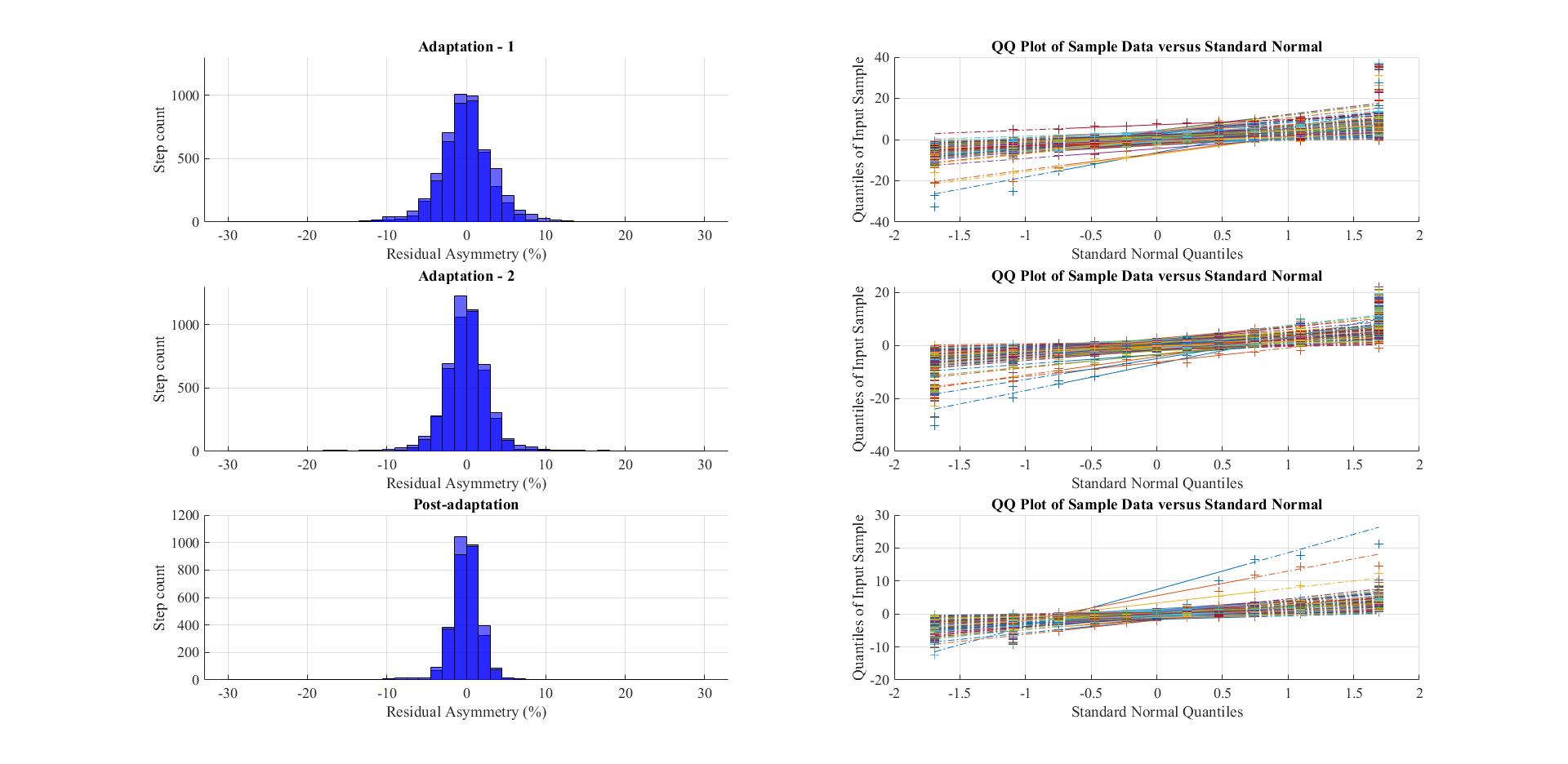

Supplement: Supplementary file 3 [file Datasheet1.zip › residual/STA/STA_T-cSI_double.jpg]

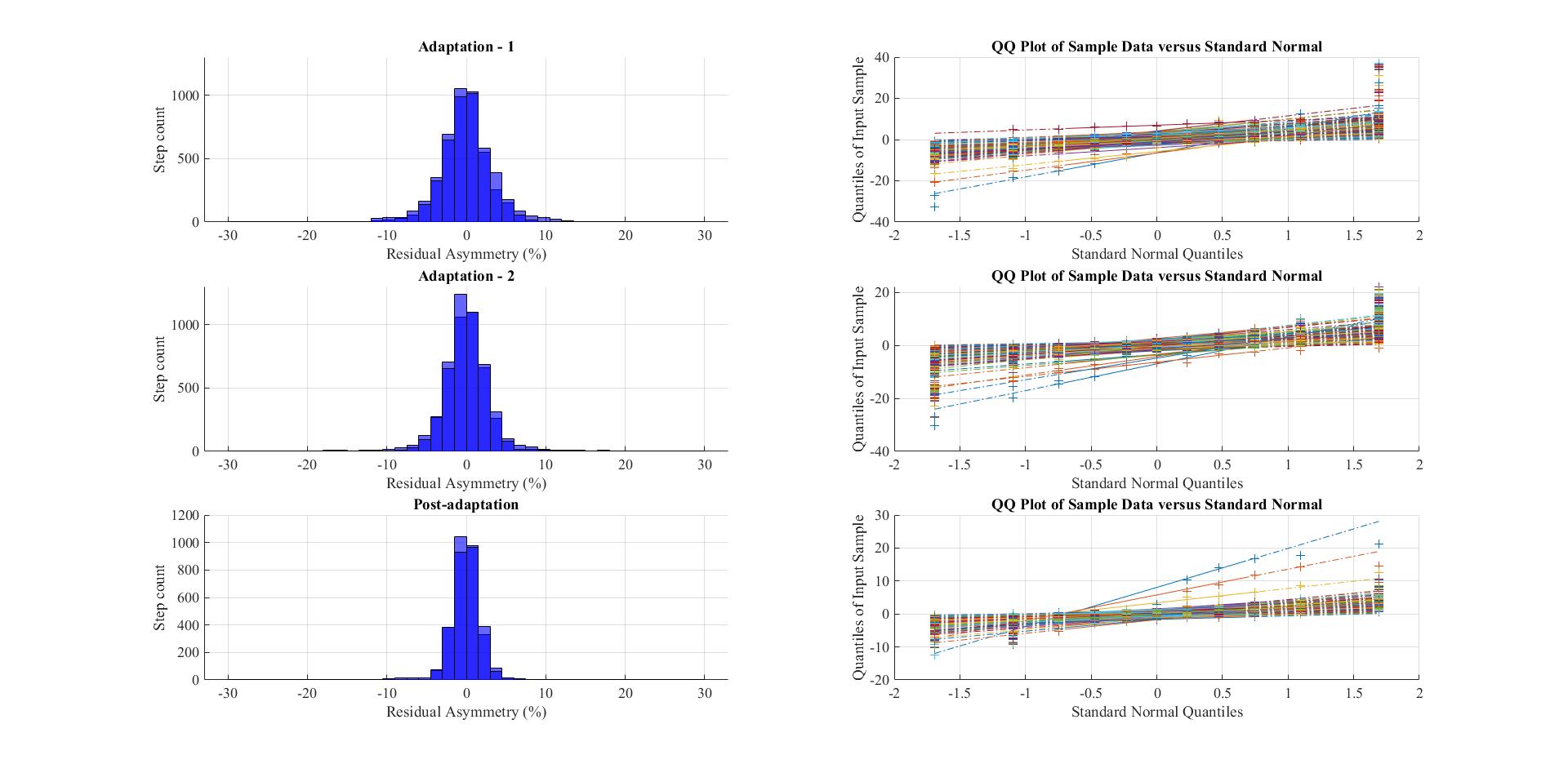

Supplement: Supplementary file 3 [file Datasheet1.zip › residual/STA/STA_T-CSI_single.jpg]

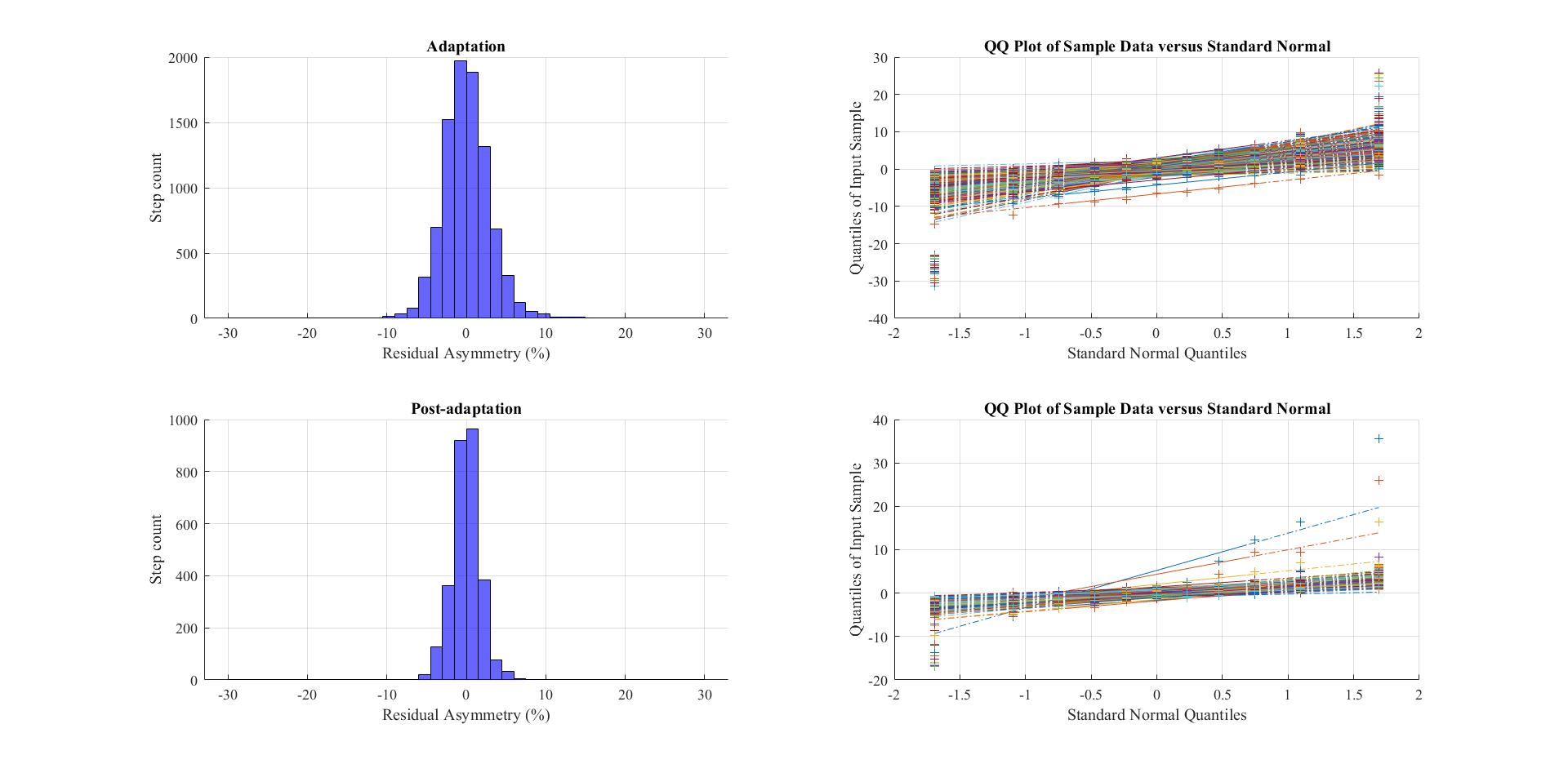

Supplement: Supplementary file 3 [file Datasheet1.zip › residual/STA/STA_T-S_double.jpg]

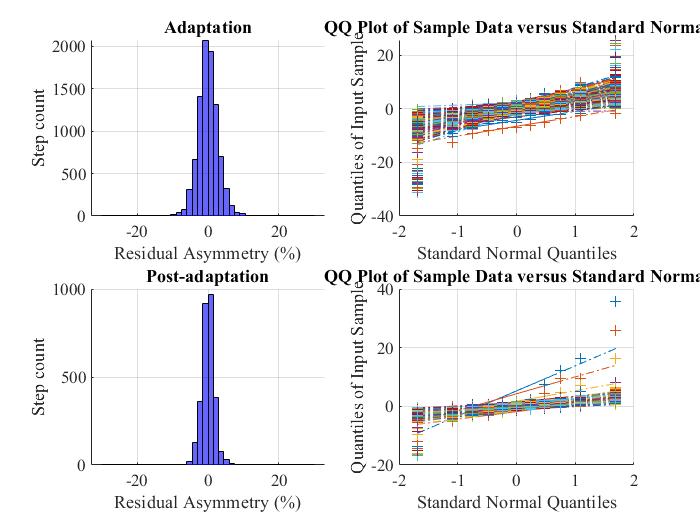

Supplement: Supplementary file 3 [file Datasheet1.zip › residual/STA/STA_T-S_single.jpg]

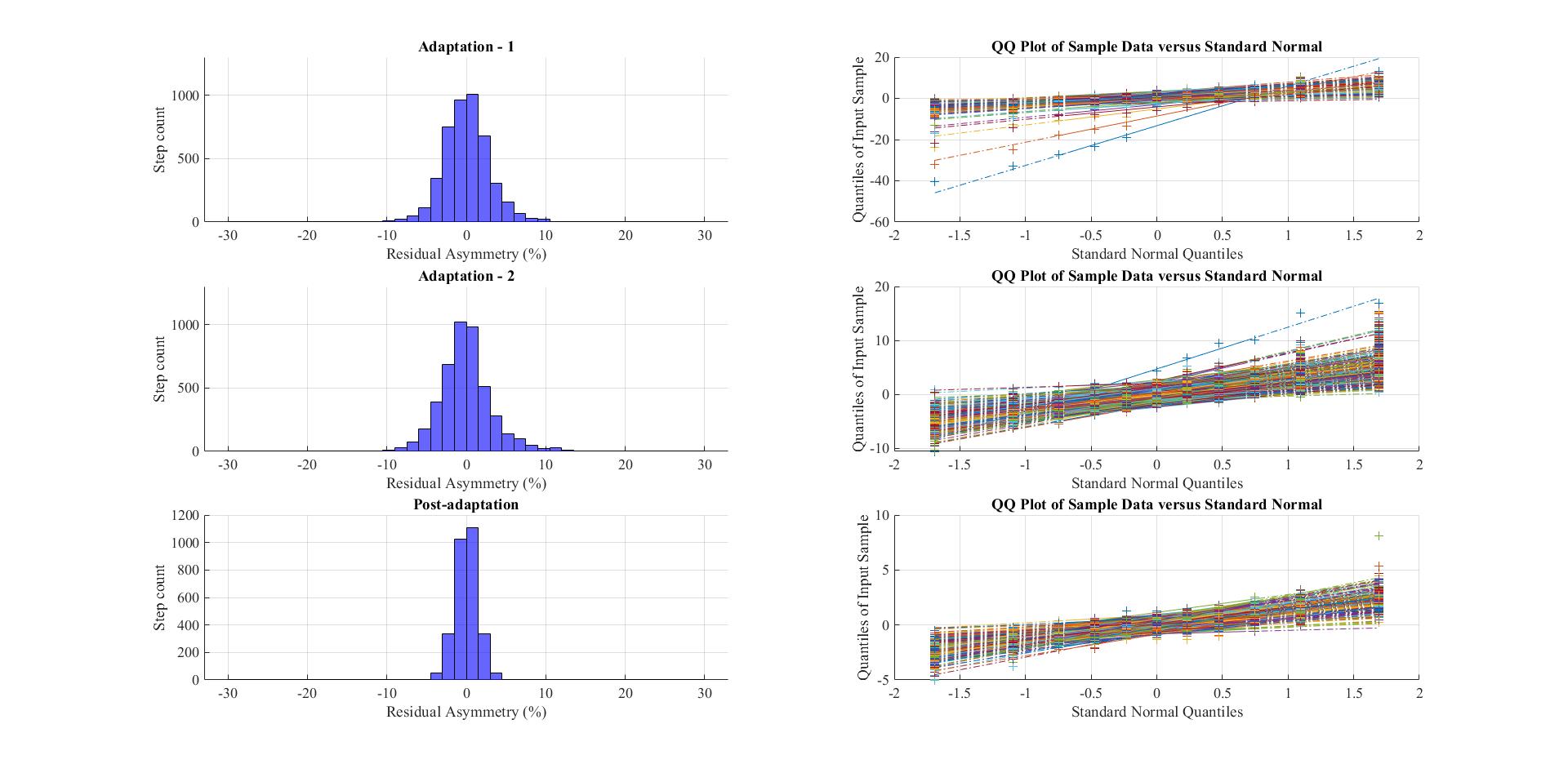

Supplement: Supplementary file 3 [file Datasheet1.zip › residual/STA/STA_T-SC_double.jpg]

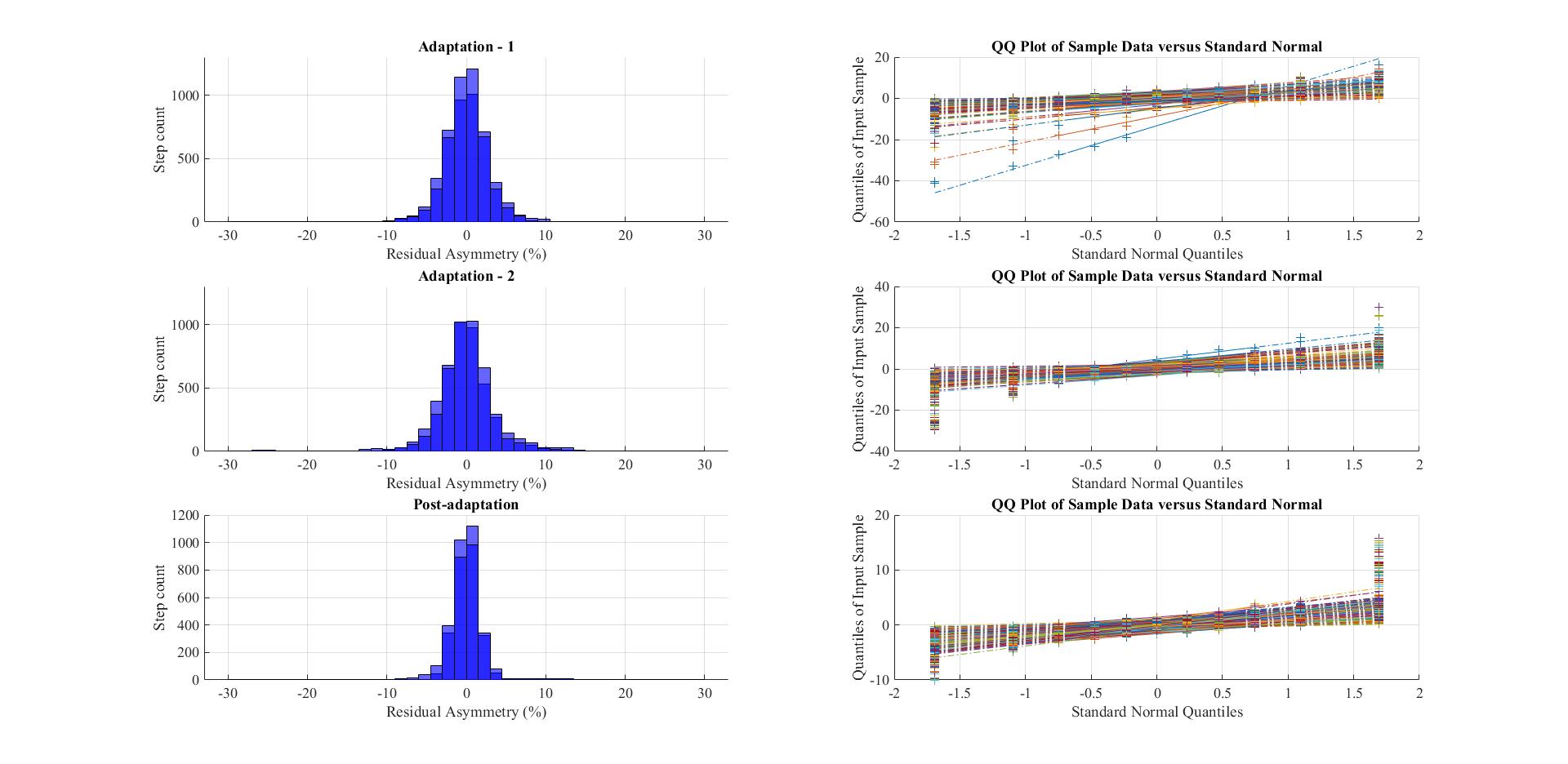

Supplement: Supplementary file 3 [file Datasheet1.zip › residual/STA/STA_T-SC_single.jpg]

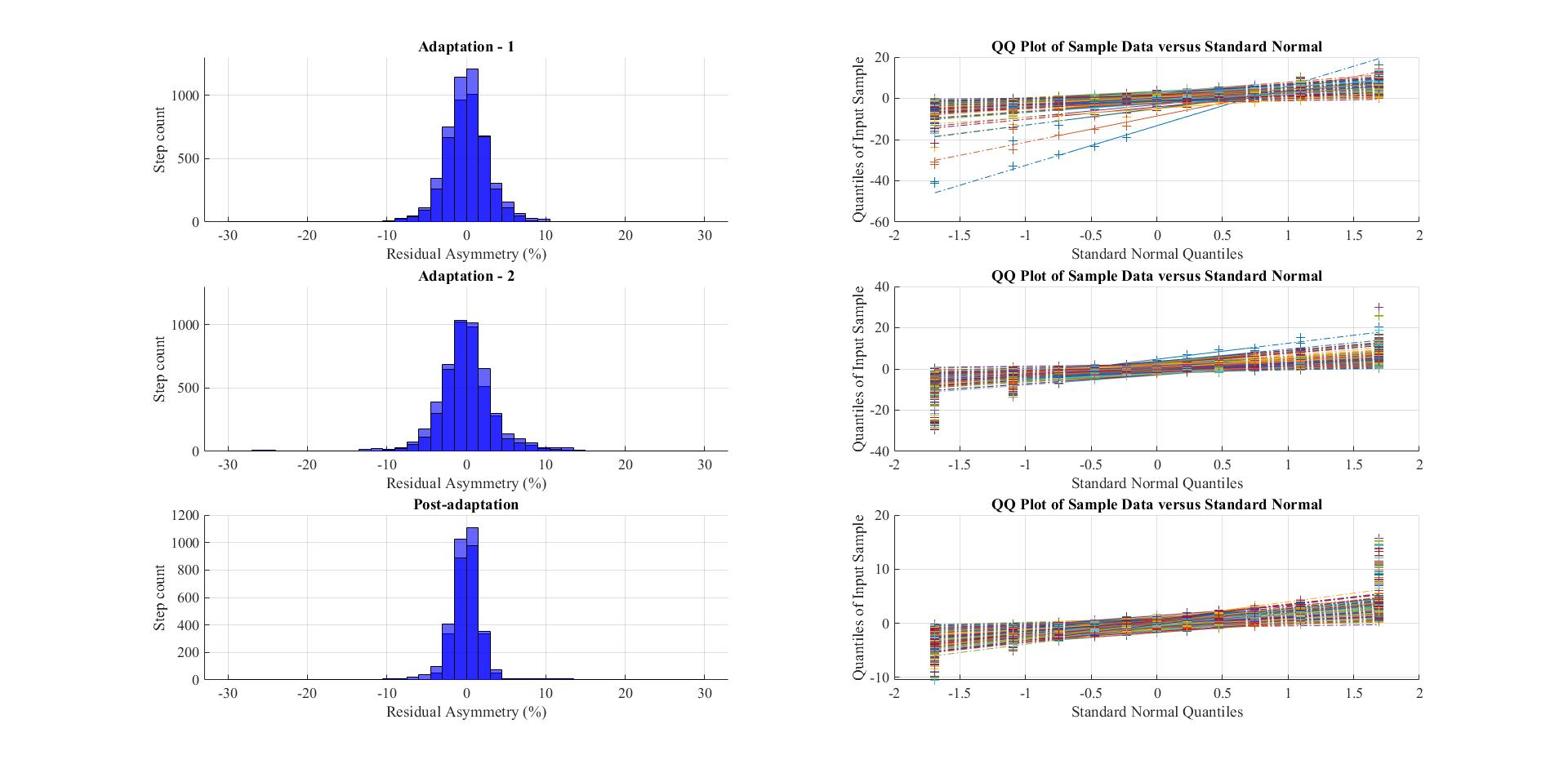

Supplement: Supplementary file 3 [file Datasheet1.zip › residual/STA/STA_T-SCI_double.jpg]

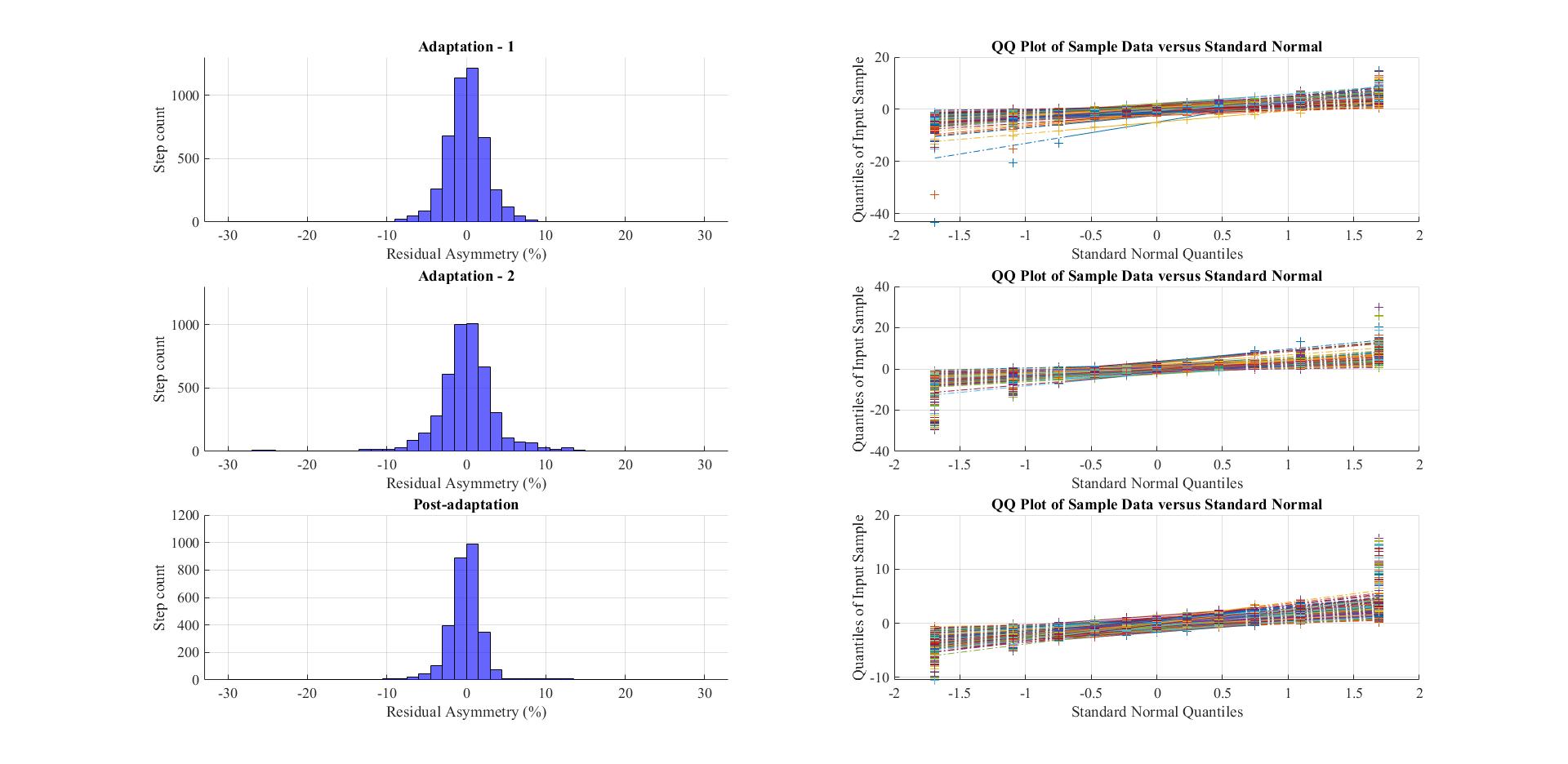

Supplement: Supplementary file 3 [file Datasheet1.zip › residual/STA/STA_T-SCI_single.jpg]

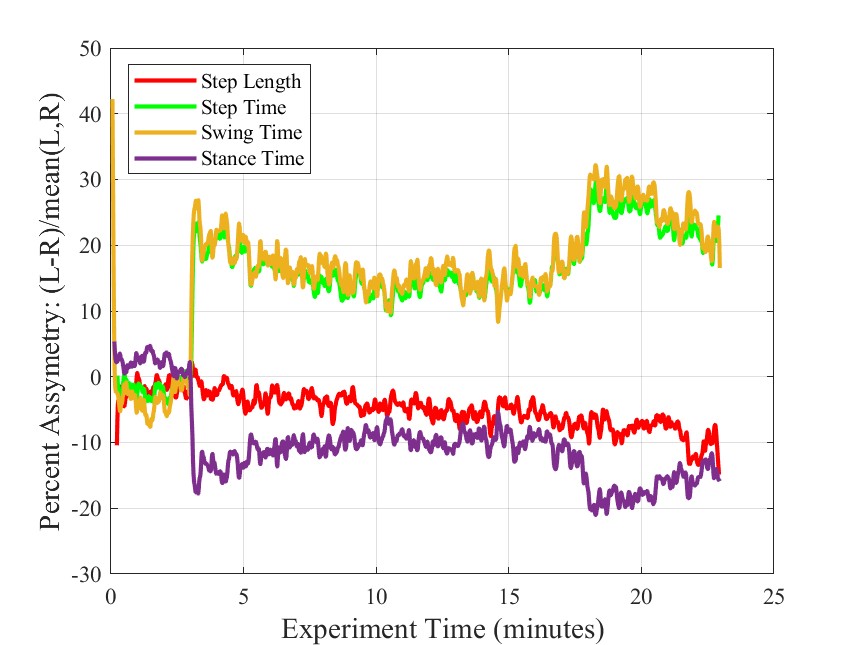

Supplement: Supplementary file 4 [file Image1.jpeg]
